# The RNA-binding proteins hnRNP H and F regulate splicing of a MYC-dependent HRAS exon in prostate cancer cells

**DOI:** 10.1073/pnas.2220190120

**Published:** 2023-07-03

**Authors:** Xinyuan Chen, Harry Taegyun Yang, Beatrice Zhang, John W. Phillips, Donghui Cheng, Frank Rigo, Owen N. Witte, Yi Xing, Douglas L. Black

**Affiliations:** ^a^Molecular Biology Interdepartmental Doctoral Program, University of California, Los Angeles, CA 90095; ^b^Bioinformatics Interdepartmental Graduate Program, University of California, Los Angeles, CA 90095; ^c^Center for Computational and Genomic Medicine, The Children’s Hospital of Philadelphia, Philadelphia, PA 19104; ^d^Department of Microbiology, Immunology, and Molecular Genetics, University of California, Los Angeles, CA 90095; ^e^Ionis Pharmaceuticals, Inc., 2855 Gazelle Ct., Carlsbad, CA 92010; ^f^Department of Molecular and Medical Pharmacology, University of California, Los Angeles, CA 90095; ^g^Jonsson Comprehensive Cancer Center, University of California, Los Angeles, CA 90095; ^h^Eli and Edythe Broad Center of Regenerative Medicine and Stem Cell Research, University of California, Los Angeles, CA 90095; ^i^Molecular Biology Institute, University of California, Los Angeles, CA 90095; ^j^Department of Pathology and Laboratory Medicine, University of Pennsylvania, Philadelphia, PA 19104

**Keywords:** alternative pre-mRNA splicing, post-transcriptional regulation, prostate cancer, MYC, HRAS

## Abstract

The MYC proto-oncogene contributes to the pathogenesis of more than half of human cancers. Malignant transformation by MYC transcriptionally up-regulates the core pre-mRNA splicing machinery and causes misregulation of alternative splicing. However, our understanding of how splicing changes are directed by MYC is limited. We performed a signaling pathway-guided splicing analysis to identify MYC-dependent splicing events. These included an HRAS cassette exon repressed by MYC across multiple tumor types. To molecularly dissect the regulation of this HRAS exon, we used antisense oligonucleotide tiling to identify splicing enhancers and silencers in its flanking introns. RNA-binding motif prediction indicated multiple binding sites for hnRNP H and hnRNP F within these cis-regulatory elements. Using siRNA knockdown and cDNA expression, we found that both hnRNP H and F activate the HRAS cassette exon. Mutagenesis and targeted RNA immunoprecipitation implicate two downstream G-rich elements in this splicing activation. Analyses of ENCODE RNA-seq datasets confirmed hnRNP H regulation of HRAS splicing. Analyses of RNA-seq datasets across multiple cancers showed a negative correlation of HNRNPH gene expression with MYC hallmark enrichment, consistent with the effect of hnRNP H on HRAS splicing. Interestingly, HNRNPF expression showed a positive correlation with MYC hallmarks and thus was not consistent with the observed effects of hnRNP F. Loss of hnRNP H/F altered cell cycle progression and induced apoptosis in the PC3 prostate cancer cell line. Collectively, our results reveal mechanisms for MYC-dependent regulation of splicing and point to possible therapeutic targets in prostate cancers.

## Significance

Transformation by the proto-oncogene MYC causes dysregulation of the pre-mRNA splicing reaction in cancer, but it is not known how mRNA isoform changes are directed by MYC. Here, we use bioinformatics to identify a splicing event in another proto-oncogene, HRAS, that is regulated by MYC across multiple tumor types. We identify splicing regulators, hnRNPs H and F, that control this HRAS exon by binding to enhancer elements within its downstream intron. Additional pan-cancer bioinformatic analyses show hnRNP H expression to be anticorrelated with MYC hallmarks, consistent with the reduced splicing of the HRAS exon in MYC-driven cancer. These findings uncover mechanisms by which MYC can alter splicing in cancer cells and provide molecular targets for potential therapeutics.

Changes in pre-mRNA splicing have emerged as important contributors to the cancer phenotype. Core splicing components including the U snRNPs assemble onto nascent RNAs to form the catalytic spliceosome that will excise each intron (1, 2). This assembly is regulated by proteins that bind to the pre-mRNA at cis-regulatory elements to direct splice site choices and create alternatively spliced mRNA isoforms (3–5). These regulators of splicing are very diverse, and each alternative splicing event is regulated by multiple factors that can act either positively or negatively on the selection of a particular isoform. Aberrant splicing in cancer can result from mutations in core spliceosomal components that give rise to aberrant mRNAs or from altered expression and modulation of regulatory RNA–binding proteins that shift the production of particular mRNA isoforms (6, 7). These changes in isoforms can affect many aspects of the tumor phenotype, including cellular growth control and cell cycle progression, suppression of apoptosis, response to hormones and growth factors, loss of cellular differentiation, metastasis, angiogenesis, and drug resistance (8). Splicing in tumor cells also appears to be more error prone, producing mRNAs that are not normally produced elsewhere and which provide appealing targets for immunotherapies (9–11).

Deregulation of the MYC proto-oncogene contributes to many cancers. MYC is a DNA-binding protein that interacts across the genome resulting in a broad deregulation of transcription, including of genes encoding components of the core splicing machinery. These changes in the levels of spliceosomal components drive cancer-associated changes in splicing, and MYC-transformed cells have been shown to be unusually sensitive to splicing inhibition (12, 13). MYC transformation also enhances expression of multiple RNA-binding regulators of splicing, leading to cancer-associated changes in alternative splicing programs (7). In glioma, neuroblastoma, and colon cancer, changes in PTBP1, hnRNP A1, and hnRNP A2 alter the splicing of pyruvate kinase and other mRNAs (14–16). In prostate cancer, changes in the expression and/or function of RNA-binding proteins such as SAM68 and hnRNP L to contribute to the cellular phenotype (17–19).

One family of RNA-binding proteins implicated in a variety of aspects of cancer are the heterogenous nuclear ribonucleoproteins H and F (hnRNP H/F). HnRNP H is encoded on three genes—H1, H2, and H3—and hnRNP F on one gene. HnRNPs H and F bind to G-run motifs GGG and GGGG, that act to enhance splicing of an alternative exon when present downstream and to repress splicing when present upstream or within the exon (20–30). Activation of splicing by G-run elements is strongly affected by regulatory elements in the upstream exon and its 3′ splice site, indicating that hnRNP H/F activity requires additional cofactors (27). The two proteins have similar effects on splicing but slightly different binding specificities and can differ in their activities on particular target exons (20, 31). They can also form a heterodimer that may allow them to coordinately affect some targets (22, 26, 29). HnRNP H was found to be up-regulated in glioma (32) as well as colon cancer, and head and neck cancers (33, 34). Oncogenic splicing switches driven by hnRNP H include targets such as IG20/MADD in glioma (32), TCF3 in lymphoma (35), HER2 and Mcl-1 in breast cancer (36, 37), KHK in hepatocellular carcinoma (38), and A-Raf in colon and head and neck cancers (33). hnRNP H also regulates alternative splicing of the oncogenic fusion transcript EWS-FLI1 (39), and the RON proto-oncogene (40), and may alter translation in glioblastoma (41). HnRNP F is less studied in the context of cancer cells but has been shown to be needed for the productive splicing of Sam68 in prostate cancer (17).

The availability of whole-transcriptome sequencing data across cancers has enabled the definition of splicing signatures in cancer tissues compared to normal cells. In an earlier study, we developed a pathway-guided transcriptomic analysis of prostate cancer using 876 RNA-seq datasets from cells ranging from normal prostatic tissue to aggressive prostate cancer (42). This identified a program of 1,039 cassette exons whose splicing correlated with MYC signaling during cancer progression. MYC-correlated exons were enriched in genes encoding splicing regulatory proteins and core spliceosomal components as well as other cellular functions. The splicing of HRAS exon 5 was found to be particularly responsive to MYC activity, and the correlation between HRAS splicing and MYC activation is found in other tumor types (42, 43). HRAS belongs to the Ras oncogene family, regulates cell division, and is involved in multiple signal transduction pathways (44, 45). HRAS exon 5 affects overall expression from the gene, such that its inclusion leads to premature translation termination, and nonsense-mediated decay (NMD) of the HRAS mRNA (46). Transcripts that escape NMD encode a C-terminal truncated p19 Ras protein with distinct functions from the canonical p21 Ras protein (47, 48). High MYC levels lead to reduced exon 5 splicing and potentially higher levels of p21 HRAS protein.

Here, we report that large-scale bioinformatic analyses of splicing and MYC expression confirm the correlation of HRAS exon 5 repression with the MYC signature score in prostate cancer and across many tumor types. To obtain mechanistic links between MYC oncogenic transformation and splice isoform choices, we dissected the regulation of HRAS exon 5 splicing. We utilized antisense oligonucleotide tiling to identify intronic splicing enhancers and silencers adjacent to the exon. RNA-binding motif enrichments indicated the presence of many hnRNP H/F-binding sites within these cis-regulatory regions. We found that both hnRNP H and F activate HRAS exon 5 splicing, and this activation required G_4_ and G_3_ elements in the downstream intron. Bioinformatic analyses of ENCODE RNA-seq datasets confirmed hnRNP H regulation of HRAS exon 5 and indicated that it is one of many exons regulated by both MYC and H/F. Additional pan-cancer bioinformatic analyses correlate the downregulation of HNRNPH expression and the upregulation of HNRNPF with the MYC hallmark score. Loss of hnRNP H/F resulted in G2/M cell cycle arrest and induced apoptosis in prostate cancer cell lines. Taken together, our results reveal mechanisms by which MYC alters splicing regulation and the phenotype of cancer cells.

## Results

### Pathway Enrichment-Guided Activity Study of Alternative Splicing (PEGASAS) Identifies HRAS Exon 5 as Repressed by MYC Transformation across Multiple Tumor Types.

**Fig. 1.**
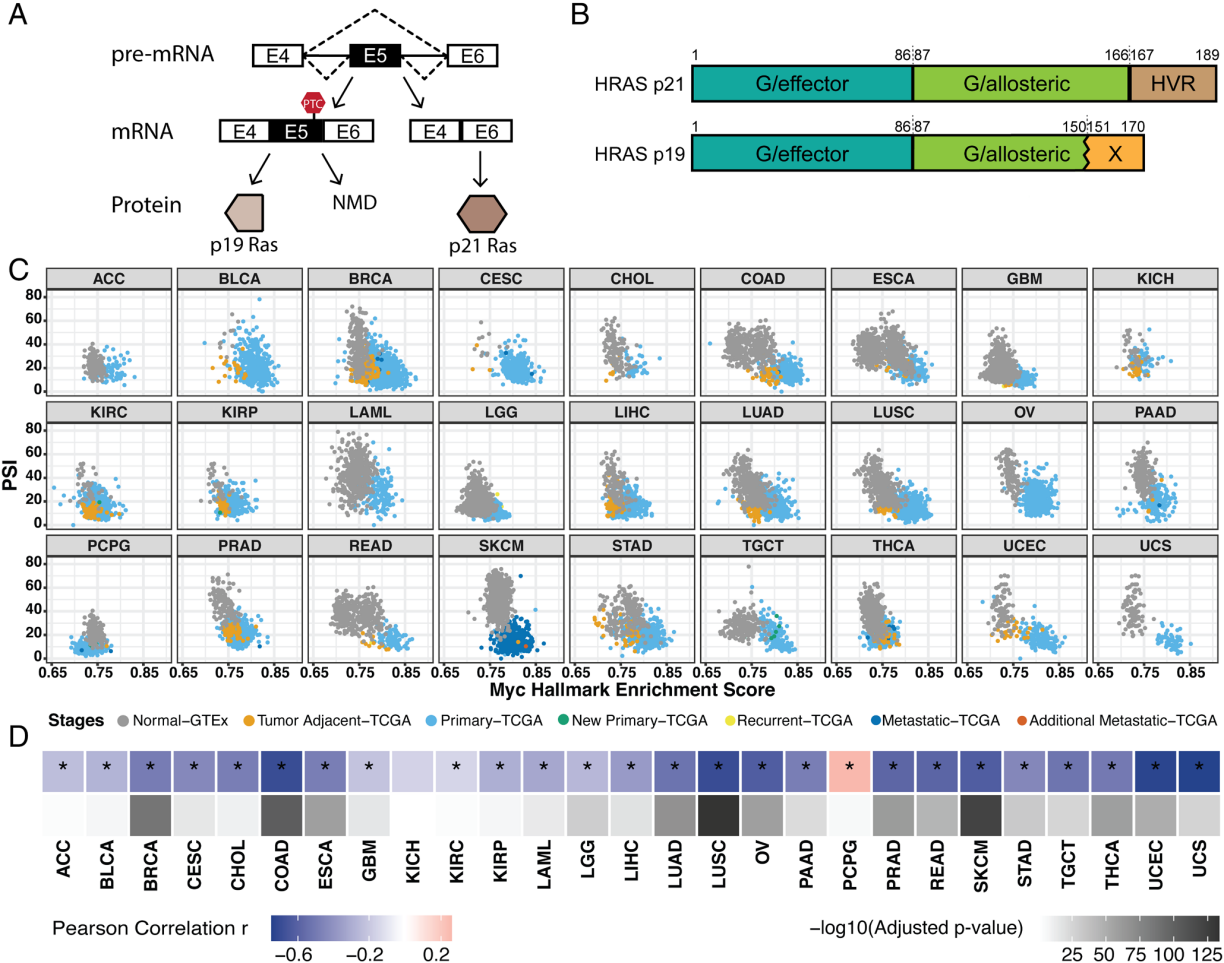
Pan-cancer analysis indicates that HRAS exon 5 is repressed by MYC activation across multiple tumor types. (*A*) Diagram of HRAS pre-mRNA alternative splicing. NMD, nonsense-mediated decay. (*B*) Domain diagram of p21 and p19 HRAS isoforms (49). G/effector, G domain/effector lobe; G/allosteric, G domain/allosteric lobe; HVR, hypervariable region. (*C*) Scatterplot matrix showing the correlation of HRAS exon 5 PSI with MYC hallmark enrichment score across multiple tumor types. ACC, adrenocortical carcinoma; BLCA, bladder urothelial carcinoma; BRCA, breast invasive carcinoma; CESC, cervical squamous cell carcinoma and endocervical adenocarcinoma; CHOL, cholangiocarcinoma; COAD, colon adenocarcinoma; ESCA, esophageal carcinoma; GBM, glioblastoma multiforme; KICH, kidney chromophobe; KIRC, kidney renal clear cell carcinoma; KIRP, kidney renal papillary cell carcinoma; LAML, acute myeloid leukemia; LGG, brain lower-grade glioma; LIHC, liver hepatocellular carcinoma; LUAD, lung adenocarcinoma; LUSC, lung squamous cell carcinoma; OV, ovarian serous cystadenocarcinoma; PAAD, pancreatic adenocarcinoma; PCPG, pheochromocytoma and paraganglioma; PRAD, prostate adenocarcinoma; READ, rectum adenocarcinoma; SKCM, skin cutaneous melanoma; STAD, stomach adenocarcinoma; TGCT, testicular germ cell tumors; THCA, thyroid carcinoma; UCEC, uterine corpus endometrial carcinoma; UCS, uterine carcinosarcoma. (*D*) Heatmap summarizing the Pearson correlation coefficient for the PSI vs. MYC score in each tumor type, accompanied by the corresponding adjusted *P*-value. *, tumor types with the statistically significant adjusted *P*-value (< 0.05).

The proto-oncogene HRAS contains a conserved poison exon (exon 5) that alters its expression and function. The exon-skipped isoform encodes the full functional p21 HRAS protein, while the exon-included isoform contains a premature termination codon (PTC) that triggers the NMD of the HRAS transcripts. Transcripts that escape from NMD are translated into a C-terminal-truncated p19 HRAS protein (46, 47) (Fig. 1*A*). HRAS p21 and p19 share most of the N-terminal G domain that mediates GTP hydrolysis (49, 50). However, p19 lacks the last 16 amino acids of the allosteric lobe and is reported not to bind GTP (47) (Fig. 1*B*). HRAS p21 also has a C-terminal hypervariable region (HVR) that is responsible for membrane binding and trafficking (50). HRAS p19 replaces this C-terminal domain with a 20-amino-acid sequence that is conserved across species but whose function is not known. Several bioinformatic studies have connected HRAS exon 5 splicing with MYC transformation. HRAS exon 5 inclusion was found to be anticorrelated with MYC activity across prostate and breast cancers (42). Greater skipping of this exon was also seen in MYC-active tumors in a pan-cancer analysis that implicated a network of SR proteins in its regulation (43).

To broadly assess HRAS splicing changes in response to MYC signaling pathway activation in tumors, we used the computational framework PEGASAS (42) to analyze RNA-seq data compiled from 5,862 tissue-matched samples from the Genotype-Tissue Expression project (GTEx) and 9,490 samples in The Cancer Genome Atlas (TCGA) (51, 52). Briefly, gene expression and exon inclusion (Percent spliced in, PSI) values were computed for all genes and exons for each sample. MYC activity scores were calculated using the MYC Targets V2 hallmark gene set from the Molecular Signatures Database (MSigDB) (53). MYC activity scores were then correlated with all exon PSI values across all the datasets. MYC activity was seen to increase with disease progression from normal tissue (gray) to tumor-adjacent benign tissue (orange), to primary tumor (light blue), and more malignant disease stages. Inclusion of HRAS exon 5 is found to negatively correlate with MYC hallmark enrichment in the majority of 27 tumor types (Fig. 1*C*). In addition to the previously observed correlation in prostate adenocarcinoma (PRAD), other epithelial cell cancers, such as colon adenocarcinoma (COAD) and lung squamous cell carcinoma (LUSC), showed particularly strong correlations (Fig. 1*D*).

### ASO Tiling Reveals Splicing Enhancers and Silencers Controlling HRAS Exon 5.

**Fig. 2.**
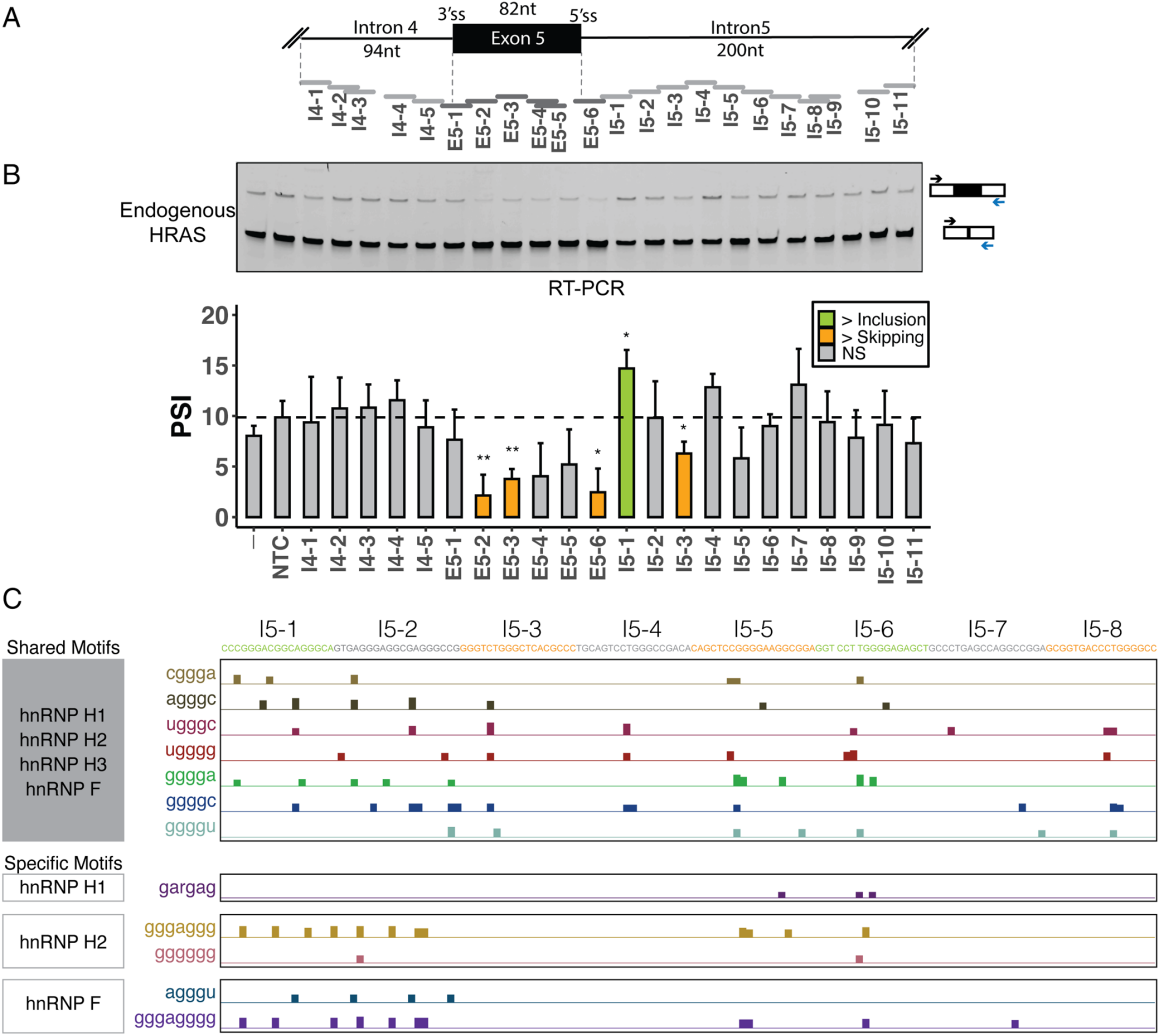
Identification of splicing cis-regulatory elements controlling HRAS exon 5. (*A*) Schematic of ASO tiling across HRAS exon 5 and its partial flanking introns, including the 376-nt region tiled by nonoverlapping 18-mer ASOs. Each horizontal bar represents an ASO. (*B*) Semiquantitative RT-PCR analyses showing the effects of ASOs on endogenous HRAS splicing. The arrows indicate RT-PCR primers for the assay of exon 5 in the endogenous HRAS transcripts. The bar graph presents the quantification of the RT-PCR calculated as percent-spliced in (PSI) (gray: control or nonsignificant; orange: more skipping; green: more inclusion). Each bar represents the mean value +/− SD of triplicates. NTC, nontargeting control. -, no-ASO mock control. NS: *P* > = 0.05; *P < 0.05; **P < 0.01 (Student’s *t*-test). (*C*) UCSC genome browser visualization of SpliceAid2 and RBPmap-predicted hnRNP H/F–binding sites on HRAS intron 5. The sequences targeted by each ASO are highlighted in orange (more skipping), green (more inclusion), and gray (no significant change). HnRNP H/F shared and specific motifs were compiled from the RBPmap and SpliceAid2 databases, respectively (55, 56).

As an approach to delineating the regulatory elements affecting exon 5, we applied antisense oligonucleotides (ASOs) that base pair and potentially block RNA elements (54). We designed and synthesized 22 ASOs that tiled across the highly conserved sequences of the HRAS exon 5 region, including 94 nucleotides (nt) of intron 4, the 82 nt exon, and 200 nt of intron 5 (Fig. 2*A*). These ASOs were 18 nt in length and were largely nonoverlapping. Four ASOs overlapped their upstream neighbor by 12 nt, 17 nt, 13 nt, and 1 nt and did not directly abut the downstream ASO (ASOs I4-3, E5-5, I5-9, and I5-11 in Fig. 2*A*). The ASOs had a uniform phosphorothioate backbone chemistry with methoxyethyl modifications at the 2′ ribose positions (2′MOE-PS). Each ASO, along with a nontargeting control (NTC), was transfected into HEK293 cells. Then, 24 h after transfection, RNA was isolated, and HRAS splicing was measured by semiquantitative RT-PCR. Comparing the exon 5 PSI value in the presence of each ASO to the NTC, we identified ASOs that significantly decrease exon 5 splicing and others that increase it (Fig. 2*B*). ASO E5-6 targeting the 5′ splice site strongly inhibited splicing, as did ASOs targeting the body of the exon, indicating the presence of exonic splicing enhancers within the HRAS exon. ASOs targeting downstream intron 5 showed diverse effects with some increasing and others decreasing exon 5 splicing, suggesting the presence of multiple intronic splicing silencers and enhancers. ASO I5-1 significantly increased exon inclusion from the 10% seen with the NTC to 14.8% and may block an intronic splicing silencer element (ISS). In contrast, ASOs I5-3 reduced exon inclusion to 6.4% and suggests the presence of a splicing enhancer in this region. Other ASOs did not induce changes that passed tests for significance.

We next constructed an HRAS minigene reporter by cloning the genomic region spanning exon 5, including the flanking introns and portions of exons 4 and 6, into the pcDNA3.1(+) expression vector (*SI Appendix*, Fig. S1*A*). We introduced an in-frame ATG start codon downstream from the CMV promoter and a TGA stop codon upstream of the BgH polyadenylation site to reduce NMD of the product mRNA. In HEK293 cells, transcripts from the minigene show 18.9% exon 5 inclusion compared to 8.0% inclusion in the endogenous HRAS transcripts and may provide a more sensitive assay for modulators of exon 5 splicing (*SI Appendix*, Fig. S1 *B* and *C*). In cotransfection experiments, we found that ASOs had pronounced effects on splicing the HRAS minigene. Consistent with their effects on endogenous HRAS transcripts, ASOs targeting the exon were strongly inhibitory, and ASO I5-1 strongly enhanced splicing. Interestingly, ASO I5-6, which had limited effect on the endogenous transcripts, also stimulated exon 5 splicing. ASOs causing exon skipping in the minigene included I4-4, I4-5, and E5-1, which block the 3′ splice site or branchpoint region. Splicing inhibition was also seen with ASOs I5-5 and I5-8 to I5-11 (Fig. 2*B* and *SI Appendix*, Fig. S1*C*). (Note that I5-8 and I5-9 overlap by 13 nt.) ASO I4-1 targeting the 5′ splice site of the upstream exon 4 generated a new band in the RT-PCR that was confirmed by sequencing to contain the retained intron 4 (*SI Appendix*, Fig. S1*D*).

Differential effects of ASOs on endogenous and minigene RNA have been reported previously (57) and may result from a number of factors. Different rates of transcription and patterns of RNA folding for the native and transfected genes could affect the ability of ASOs to act on the RNA. Also, mature endogenous RNA is already present at the time of ASO transfection and may not turn over completely during the assay of the ASOs. In contrast, the minigene is cotransfected with the ASOs, so all RNA is processed in their presence. The splicing changes seen in the native RNA may also be dampened by the low baseline level of exon inclusion (8.0%) seen in the endogenous transcripts.

A previous study identified a splicing silencer element called rasISS1 in HRAS intron 5 (58) (Fig. 4*A*). The inhibitory sequence of rasISS1 maps to the region covered by ASOs I5-1 and I5-2. Our data also indicate a silencer in the I5-1 region. The limited effect we observe from I5-2 may be due to the secondary structure proposed for this region interfering with targeting by antisense oligo (58). We have focused on regulatory elements where ASOs inhibited splicing of either the endogenous HRAS exon (I5-3) or the minigene exon (I5-5 and I5-8/9), indicating the presence of intronic splicing enhancer elements.

To identify trans-acting factors that potentially bind the HRAS splicing regulatory elements, we examined the sequences surrounding HRAS exon 5 with the motif-finding tools RBPmap and SpliceAid2 (55, 56). The two programs use different motif definitions for RNA-binding proteins. The combined results identified many binding motifs for hnRNP H and its paralog hnRNP F downstream of exon 5, as well as motifs for hnRNP A1, SRSF5, and other proteins (Fig. 2*C* and *SI Appendix*, Fig. S2 *A* and *B*). Previous work identified hnRNP A1 as a repressor of exon 5 that binds to the rasISS1 element (58). The SR proteins SRSF2 and SRSF5 were also identified as factors that correlate with exon 5 activation (58). We decided to focus on hnRNP H and hnRNP F and to assess their effects on the HRAS exon 5.

### HnRNPs H and F Activate HRAS Exon Splicing.

Heterogenous nuclear ribonucleoproteins H and F (H1, H2, H3, and F) are paralogous splicing factors that activate splicing when bound downstream of alternative exons. H and F both bind to G-run motifs GGG and GGGG, although they exhibit slightly different binding specificities (20, 22–26, 29). HnRNP H3 is a pseudogene that lacks the N-terminal RNA recognition motif (59, 60). HnRNPs H1 and H2 are highly similar in peptide sequence (61) but differ in their relative expression, with H1 more highly expressed in most cell types including HEK293 cells and a MYC-dependent prostate cancer model (42, 51). We have focused on hnRNP H1 and F.

**Fig. 3.**
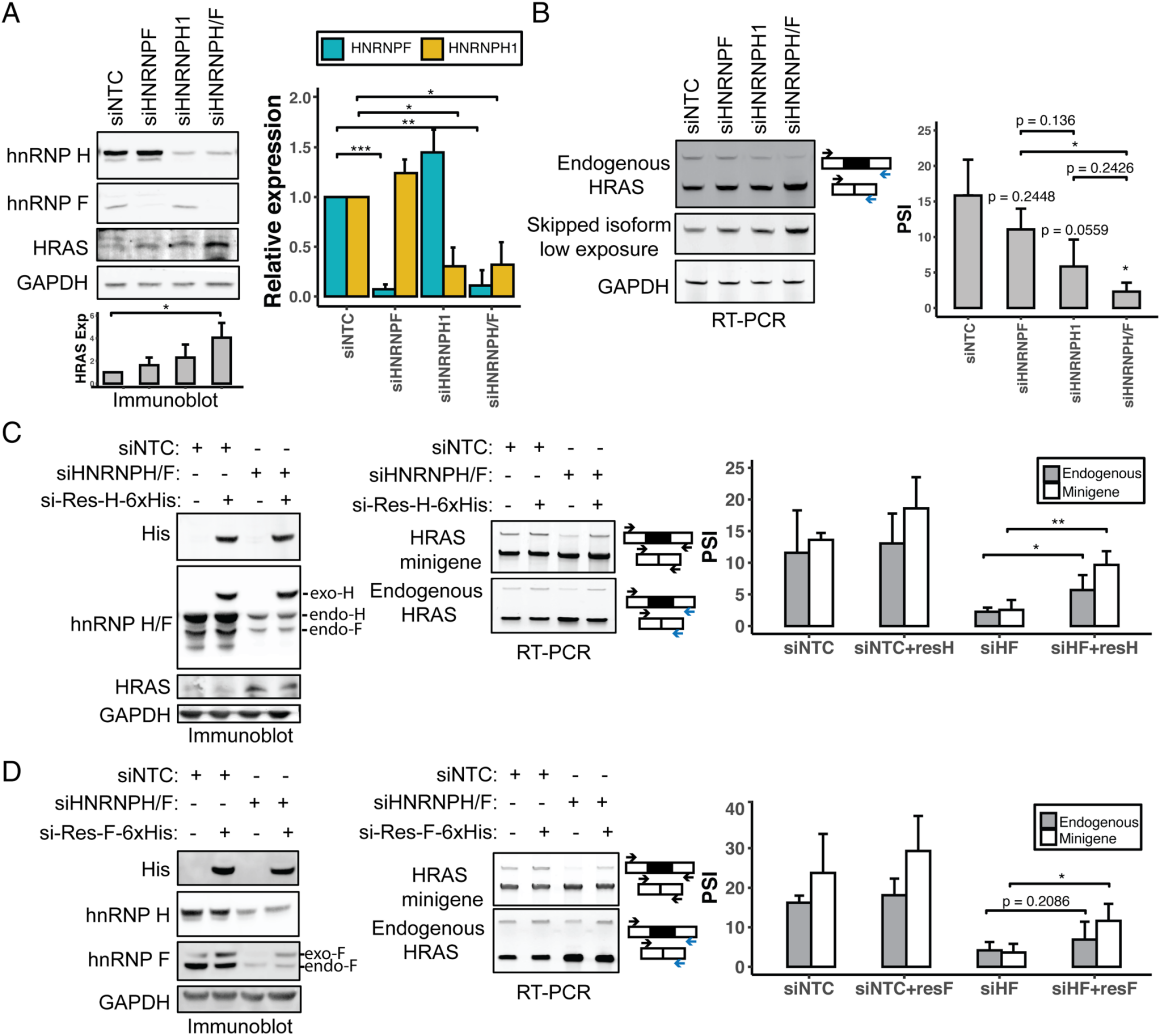
HnRNP H and F activate HRAS exon 5 splicing. (*A*) Immunoblot showing the expression of hnRNP H, hnRNP F, and HRAS proteins in HEK293 cells, after transfection with control, HNRNPF, HNRNPH1, or HNRNPH/F siRNAs. GAPDH was used as a loading control. The bar graph (*Right*) shows the quantification of hnRNP H and F proteins in response to each siRNA perturbation. The grayscale bar graph (*Bottom*) shows the quantification of HRAS p21 protein expression in response to each siRNA perturbation. (*B*) RT-PCR analysis endogenous HRAS splicing after siRNA knockdown of hnRNPs H and F. The bar graph (*Right*) shows the quantification of the RT-PCR results. (*C* and *D*) Rescue of hnRNP H/F expression after knockdown in HEK293 cells. Immunoblot of hnRNP H, hnRNP F, His-tagged rescue protein, and HRAS in HEK293 cells. Cells were transfected with control or HNRNPH/F siRNAs followed by transfection with siRNA-resistant C-terminal 6xHis–tagged hnRNPs H (*C*) or F (*D*). RT-PCR of minigene and endogenous HRAS splicing in each experimental condition is quantified in the bar graphs (*Right*). Each bar represents the mean +/− SD of triplicates. NS: *P* > = 0.05; *P < 0.05;**P < 0.01; ***P < 0.001 (Student’s *t*-test).

To assess the effects of hnRNPs F and H on HRAS splicing, we performed siRNA-mediated knockdown in HEK293 cells. This used siRNAs that target either the F or H1 transcript alone or an siRNA that targets a conserved sequence found in both transcripts (23). Immunoblot confirmed that the siRNAs depleted hnRNP F by 93% and H1 by 70% when they were targeted individually and by 89% and 68% when hnRNPs F and H were targeted together (Fig. 3*A*). Depletion of F or H individually led to a modest increase in the other factor, an apparent cross-regulation that is commonly observed for paralogous pairs of RNA-binding proteins. Then, 72 h after introduction of the siRNAs, we assayed the splicing of exon 5 in the endogenous HRAS transcripts by RT-PCR. Depletion of either hnRNP F or hnRNP H decreased exon 5 splicing, with a stronger effect seen with the loss of hnRNP H, despite its less complete depletion. The dual-targeted siRNA had a similar effect to that targeting hnRNP H alone. Thus, both H and F act to enhance HRAS splicing with H being the stronger regulator (Fig. 3*B*). H/F knockdown resulted in an upregulation of the HRAS p21 protein isoform as seen by immunoblot, consistent with the observed splicing changes (Fig. 3*A*). Both the siRNAs targeting hnRNP H led to reduced exon 5 splicing, indicating that the changes in splicing are likely not due to off-target effects of the siRNAs. To further rule out off-target effects, we reexpressed 6xHis-tagged siRNA-resistant HNRNPH1 or HNRNPF cDNAs after siRNA depletion of the endogenous transcripts (Fig. 3 *C* and *D* and *SI Appendix*, Fig. S3*A*). Immunoblot confirmed the expression of recombinant hnRNP H or F at levels comparable to the endogenous proteins. Reexpression of either hnRNP H or F stimulated splicing of HRAS exon 5 in both minigene and endogenous RNAs, thus validating hnRNP H and F as splicing activators and ruling out off-target effects of the siRNAs.

### G_3_ and G_4_ Elements in the Downstream Intron Mediate the hnRNP H/F–Dependent Enhancement of HRAS Splicing.

**Fig. 4.**
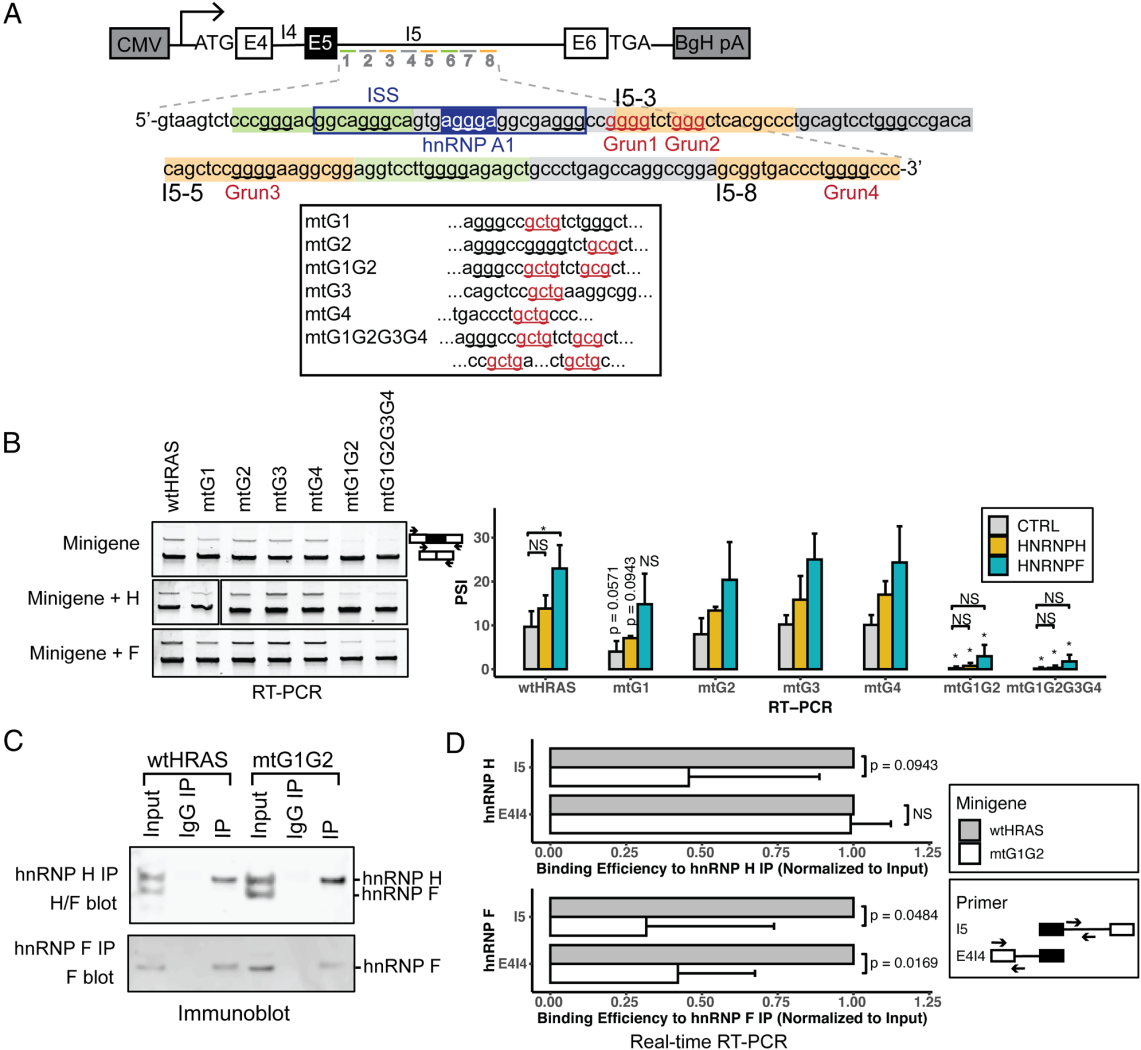
HnRNP H and hnRNP F modulate HRAS exon 5 splicing through G-run elements within the downstream ISE. (*A*) Diagram of HRAS minigene reporters carrying mutations at putative hnRNP H/F–binding motifs. Intron 5 nucleotide sequences targeted by ASOs I5-1 to I5-8 are shown in orange as potential enhancers and green as potential suppressers. G-runs within this intron 5 region are underlined, and those that are within the enhancer regions and mutated are labeled in red and numbered. Mutations in these elements are indicated below, with the mutated sequences underlined and labeled in red. The known silencer element rasISS1 is boxed in navy, and its putative hnRNP A1–binding motif is highlighted (58). (*B*) RT-PCR analyses showing the splicing changes of wild-type and mutant minigene reporters after cotransfection with control (*Top*), hnRNP H (*Middle*), and hnRNP F (*Bottom*) expression plasmids (wt, wild type; mt, mutant). The bar graph presents the quantification of RT-PCR results, with the mean +/− SD of triplicates. The *P*-values for the changes between wtHRAS and the mtG1, mtG1G2, and mtG1G2G3G4 mutants are indicated above the bars, where NS indicates *P* > = 0.05; and * indicates *P* < 0.05 (Student’s *t*-test). Horizontal bars indicate p-values for changes between control and hnRNP H or F expression. (*C*) Immunoblot of hnRNP H and hnRNP F immunoprecipitates. HEK293 cells transfected with wtHRAS or mtG1G2 minigenes were immunoprecipitated using anti-hnRNP H or anti-hnRNP F antibodies, or nonimmune IgG. The immunoblot shows the recovery of hnRNP H, hnRNP F, or both in the precipitate. (*D*) RNA immunoprecipitation assayed by real-time RT-PCR of wild-type and G1G2 mutant HRAS intron 5 RNA bound by hnRNP H and hnRNP F proteins. The bar graphs plot the recovered RNA in hnRNP H and F immunoprecipitates quantified using two sets of primers: The I5 product targets an intronic region neighboring the mutation, and the E4I4 product targets the intron upstream. The recovered RNA in each IP was normalized to its input control. The mtG1G2 RNA was then measured relative to the wtHRAS RNA normalized to one. The bars show the mean +/− SD of duplicates.

HnRNPs H and F bind to motifs containing runs of three or four G nucleotides which act as splicing enhancers when found in a downstream intron (22, 29). There are ten G-runs downstream of exon 5, of which four were identified by the ASO tiling as enhancer elements (Fig. 4*A*). We constructed a series of minigene reporters carrying single mutations at each of these four G-run elements, a double mutation of the neighboring G1 and G2 runs, and a mutation of the four G-runs (G1, G2, G3, G4). After transfection into HEK293 cells, the splicing of each of these constructs was compared to the wild-type clone by RT-PCR (Fig. 4*B*). We observed small decreases in exon 5 splicing when either G1 or G2 was mutated and minimal splicing changes resulting from G3 or G4 mutations. In the constructs carrying single G-run mutations, the expression of recombinant H or F enhanced exon inclusion (Fig. 4*B* and *SI Appendix*, Fig. S3*B*). The double mutation of both G1 and G2 resulted in a nearly complete loss of exon 5 splicing, and a similar effect was seen when all four G runs were mutated together. Notably, for these mtG1G2 and mtG1G2G3G4 constructs, the overexpression of H or F could no longer rescue exon 5 splicing. A slight increase in exon 5 inclusion over the baseline for these mutants may result from binding to additional hnRNP H/F motifs within this complex regulatory region. We also tested mutations in other G-runs downstream of exon 5, where either the blocking ASOs indicated the presence of a splicing enhancer or in some cases had minimal effect (G-runs located in the I5-1, I5-4, and I5-6 targeted regions; Fig. 4*A* and *SI Appendix*, Fig. S3*C*). The splicing changes induced by these mutations were mostly consistent with the ASO data, except the second G-run in the I5-1 region, where mutation indicated that it acted as an enhancer but the ASO was apparently blocking a silencer (*SI Appendix*, Fig. S3*C*). This region is thus complex and likely contains multiple regulatory elements, one of which might be an additional hnRNP H/F–dependent enhancer. Altogether, the results indicate that multiple G-runs within HRAS intron 5 act as hnRNP H/F–dependent splicing enhancers, with individual elements acting redundantly and with G1 and G2 having the strongest effects.

To assess the interactions of hnRNPs H and F with HRAS intron 5, we assayed for the presence of HRAS RNAs in hnRNP H/F immunoprecipitates. HEK293 cells were transfected with the wild-type HRAS minigene or that carrying the G1G2 mutation. Endogenous hnRNPs H and F were immunoprecipitated with antibodies reactive with each protein. The specificity of the pull-downs and yield of the immunoprecipitations were monitored by immunoblot (Fig. 4*C*). RT-PCR analyses of the immunoprecipitated RNA identified HRAS pre-mRNA associated with the H and F immunoprecipitates but not the control IgG. Primer pairs were designed to amplify either the exon4-intron4 junction or a segment of intron 5 neighboring the G-runs. These yielded the expected products from the minigene reporter whose unspliced products were much more abundant than the nascent endogenous HRAS RNA. A minus reverse transcriptase control confirmed the absence of minigene DNA (*SI Appendix*, Fig. S3*D*). To quantify the amounts of mutant and wild-type intron 5 RNAs in each pulldown, we performed qRT-PCR, normalizing the amount of intron 5 RNA in the precipitates to the input. Precipitated RNAs and input RNAs were amplified with intron 5 primers or with exon4-intron4 primers. Since the total amount of each RNA region could vary with the splicing level, we normalized each IP to the total RNA in the sample and measured the amount recovered in each IP as a fraction of the total with the wild-type RNA normalized to 1. We found that the binding of HRAS RNA to hnRNPs H and F was substantially reduced by G1G2 mutation (Fig. 4*D*). The intron 5 fragment was more abundant in the immunoprecipitates than the exon 4–intron 4 fragment, and this binding was substantially reduced by the G1G2 mutation. The G1G2 mutation also led to reduced binding of the exon 4–intron 4 fragment to hnRNP F. This may be due to additional hnRNP F–binding motifs that are dependent on the G1G2 interaction. Alternatively, hnRNP F may only bind to the G1G2 region, while hnRNP H binds additional sites within the E4-I4 fragment—indicating differences in the binding preferences of hnRNPs H and F. Such differences would explain the higher activity of hnRNP H in regulating HRAS splicing. Overall, the data indicate that hnRNPs H and F interact with the G-runs in HRAS intron 5 to activate exon 5 splicing.

### HNRNPH Gene Expression Decreases with MYC Activation across Multiple Tumor Types.

**Fig. 5.**
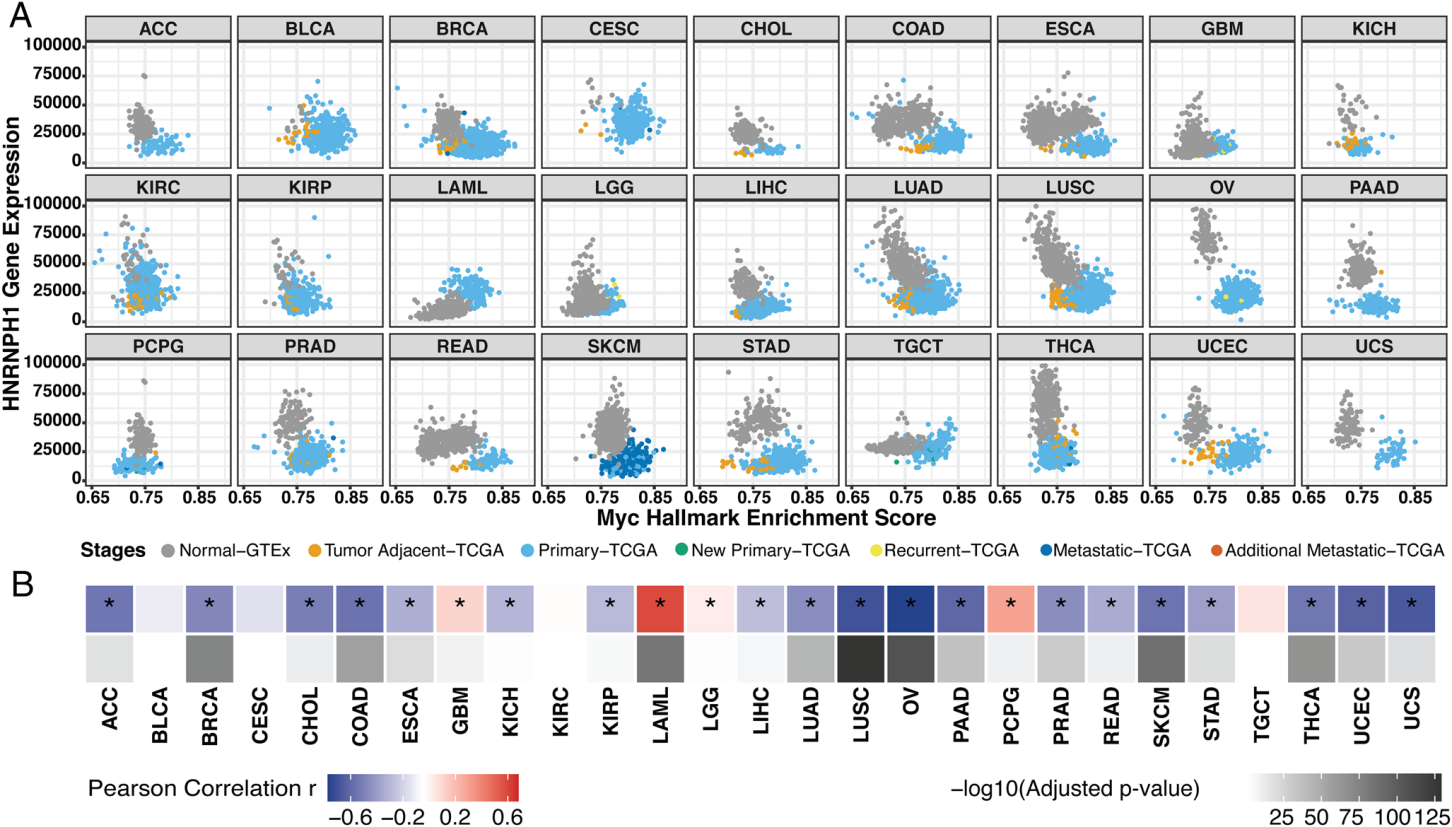
HNRNPH1 gene expression decreases with MYC activation across tumor types. (*A*) Scatterplot matrix showing the correlation of normalized HNRNPH1 versus MYC hallmark enrichment score across the disease spectrum in multiple tumor types. (*B*) Heatmap summarizing the Pearson correlation coefficient of MYC vs. HNRNPH1 expression for each tumor type, accompanied by its adjusted *P*-value. *, tumor types with the statistically significant adjusted *P*-value (< 0.05).

To more broadly assess the association of hnRNPs H and F with MYC, we performed a correlation analysis of the MYC activity score and normalized splicing factor expression across tumor types. We used DEseq2 to normalize the read counts of 220 genes encoding splicing factors (62) in the TCGA and GTEx samples analyzed above. We then correlated splicing factor expression with MYC activity scores computed from PEGASAS. HNRNPH1 exhibited a clear negative correlation with MYC activation in the majority of 27 tumor types, including prostate cancer (PRAD, Fig. 5 *A* and *B*). In contrast, HNRNPF exhibited a positive correlation with MYC activation in almost all tumor types (*SI Appendix*, Fig. S4 *A* and *B*). The negative correlation between HNRNPH1 and MYC was found in multiple epithelial cancers such as breast invasive carcinoma (BRCA), COAD, lung squamous cell carcinoma, and ovarian serous cystadenocarcinoma. Interestingly, the correlations were reversed in the acute myeloid leukemia (LAML) samples. In these tumors, HNRNPH1 was positively correlated with MYC and HNRNPF negatively correlated. Additional analyses showed that HRAS exon 5 PSI is positively correlated with HNRNPH1 gene expression across tumor types and negatively correlated with HNRNPF expression (*SI Appendix*, Fig. S5 *A*–*D*). These findings agree well with our results identifying that hnRNP H1 is an activator of HRAS exon 5 splicing.

### The HRAS Exon Is One of Many Exons Controlled by Both MYC and hnRNP H/F Activity.

**Fig. 6.**
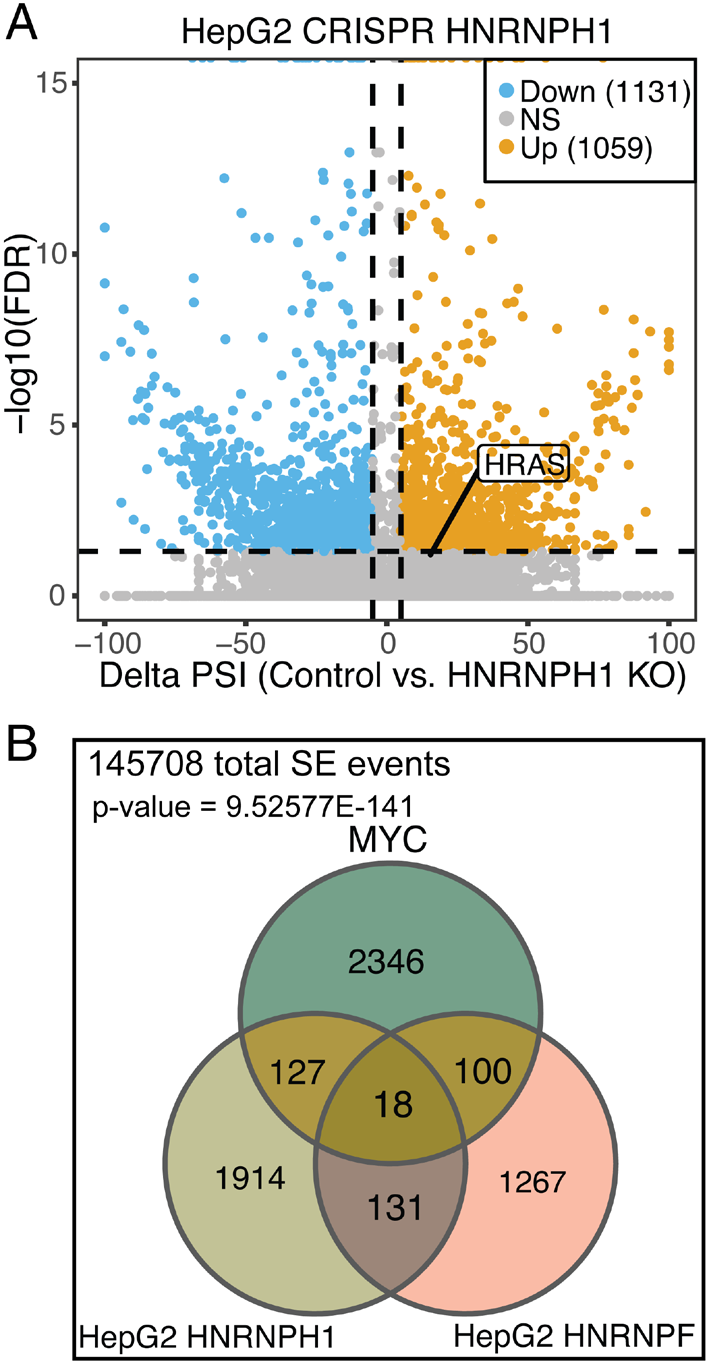
HRAS exon 5 is one of many exons controlled by both MYC and hnRNPs H and F. (*A*) Scatterplot showing the SE (Skipped Exon) events detected by RNA-seq from the ENCODE project in HepG2 cells after HNRNPH1 CRISPR knockout. Significant SE events were filtered by junction reads per event > 10, |deltaPSI| > 0.05, and FDR < 0.05. (*B*) Venn diagram showing the overlapping significant SE events across three datasets: MYC on vs. MYC off, HNRNPH1 KO vs. Control, and HNRNPF KO vs. Control. The numbers reflect overlapping events showing changes in each comparison without considering the direction of the changes.

To identify additional exons regulated by hnRNPs H and F, we analyzed RNA-seq datasets from the ENCODE project (63). Using rMATS-turbo, we compared RNA from HepG2 cells after HNRNPH1 or HNRNPF knockout to RNA from the nontargeted control cells (Fig. 6*A* and *SI Appendix*, Fig. S6*A*) (64). This identified 2190 and 1516 changes in skipped exons (SE) after H and F knockout, respectively. HnRNP H can act either as a splicing repressor or activator depending on its binding location (31). Consistent with this, we observed that approximately 50% of the exons showed reduced splicing upon HNRNPH1 knockout, indicating that the protein acted to enhance their splicing. HRAS exon 5 exhibited a PSI of 11% in the HNRNPH1 knockout samples, compared with 27% in the nontargeted control, although the FDR value of 0.0694 just missed the cutoff of 0.05 (*SI Appendix*, Fig. S6 *B*, *Left*). Similar overall results were obtained with the HNRNPF knockout, except that HRAS exon 5 showed very limited change upon HNRNPF knockout compared with the nontargeted control that was not statistically significant. This limited effect might result from the lower expression of hnRNP F yielding a smaller effect on the PSI after knockout (*SI Appendix*, Fig. S6 *A* and *B*, *Right*). To further validate the ENCODE HepG2 findings, we knocked down hnRNPs H and F by siRNA in HepG2 cells and performed RT-PCR on the endogenous HRAS transcripts. As seen in the ENCODE RNA-seq data and in HEK293 cells (Fig. 3), the loss of H alone reduced HRAS exon inclusion in HepG2 cells, and the double knockdown had a stronger effect (*SI Appendix*, Fig. S6*C*). Interestingly, in these cells, the double depletion of H and F also significantly reduced MYC expression as seen by immunoblot (see below). The data for HepG2 and HEK293 both indicate that hnRNP H1 is an activator of HRAS exon 5 splicing (Fig. 3*B*).

Analyses of the other ENCODE line, K562, also showed that HRAS exon 5 was positively regulated by hnRNP H1 (*SI Appendix*, Fig. S6*D*). However, in these cells, hnRNP F gave different results exhibiting an increase in exon 5 splicing after HNRNPF knockdown (*SI Appendix*, Fig. S6*D*). Presumably, K562 expresses a different complement of splicing regulators that allow hnRNP F to act differently on some of its target RNAs. Notably, in the pan-cancer gene expression analysis (Fig. 5 and *SI Appendix*, Fig. S4), we found that hnRNP H and F expression show the opposite correlation with MYC in acute myeloid leukemia (LAML) than what is seen in other tumor types. Thus, the lymphoblastic K562 cells derived from a chronic myelogenous leukemia may behave more similarly to LAML than to HepG2.

To examine whether other exons that are regulated by hnRNPs H and F are also regulated by MYC, similarly to HRAS, we analyzed RNA-seq data from a prostate cancer model carrying a doxycycline-inducible MYC gene (42). This identified 2,591 differentially spliced SE events between the MYC-on and MYC-off conditions. These MYC-dependent SE events overlapped with several hundred of the hnRNP H1 or F–dependent SE events from HepG2 (Fig. 6*B*: with SE events summarized in Dataset S1). A hypergeometric test comparing these exon sets yielded a *P*-value of 9.53 × E^−141^ indicating that a statistically significant fraction of the splicing events regulated by MYC are also controlled by hnRNP H1 and/or hnRNP F. Gene ontology analyses (PANTHER) (65) indicate that the genes containing splicing events in the three-way overlap are enriched for genes involved in metabolic processes, with mRNA metabolic processes as the top term (*SI Appendix*, Fig. S6*E*). Thus, there are multiple exons controlled by both MYC and hnRNPs H and F, and HRAS exon 5 is just one example.

Although significant, the number of SE events overlapping in the MYC and hnRNP H/F exon sets could be reduced by a number of factors. Notably, the MYC-controlled SE events were identified in a prostate cancer model derived from patient tissue that will exhibit very different transcriptional and splicing regulation from HepG2 cells. To further compare SE events controlled by MYC with those controlled by other RNA-binding proteins, we ran pairwise comparisons between MYC-controlled SE events and additional splicing factor–controlled SE events, identified in CRISPR RNA-seq data in HepG2 cells from ENCODE. For each pairwise comparison, we determined the Jaccard Index of the intersect over the union of the two exon sets. The Jaccard Indices for hnRNP H1 plus MYC and hnRNP F plus MYC were higher than multiple other RBPs (*SI Appendix*, Fig. S6*F*). The only factors observed with higher Jaccard Indices were SRSF7, which is a known MYC target (43), and the core spliceosome subunit SF3A1. This analysis further underscores the relationship between the MYC and hnRNP H/F controlled splicing programs, with the HRAS exon 5 as one example.

### HnRNP H/F Are Required for Cell Proliferation in Prostate Cancer Cell Lines.

**Fig. 7.**
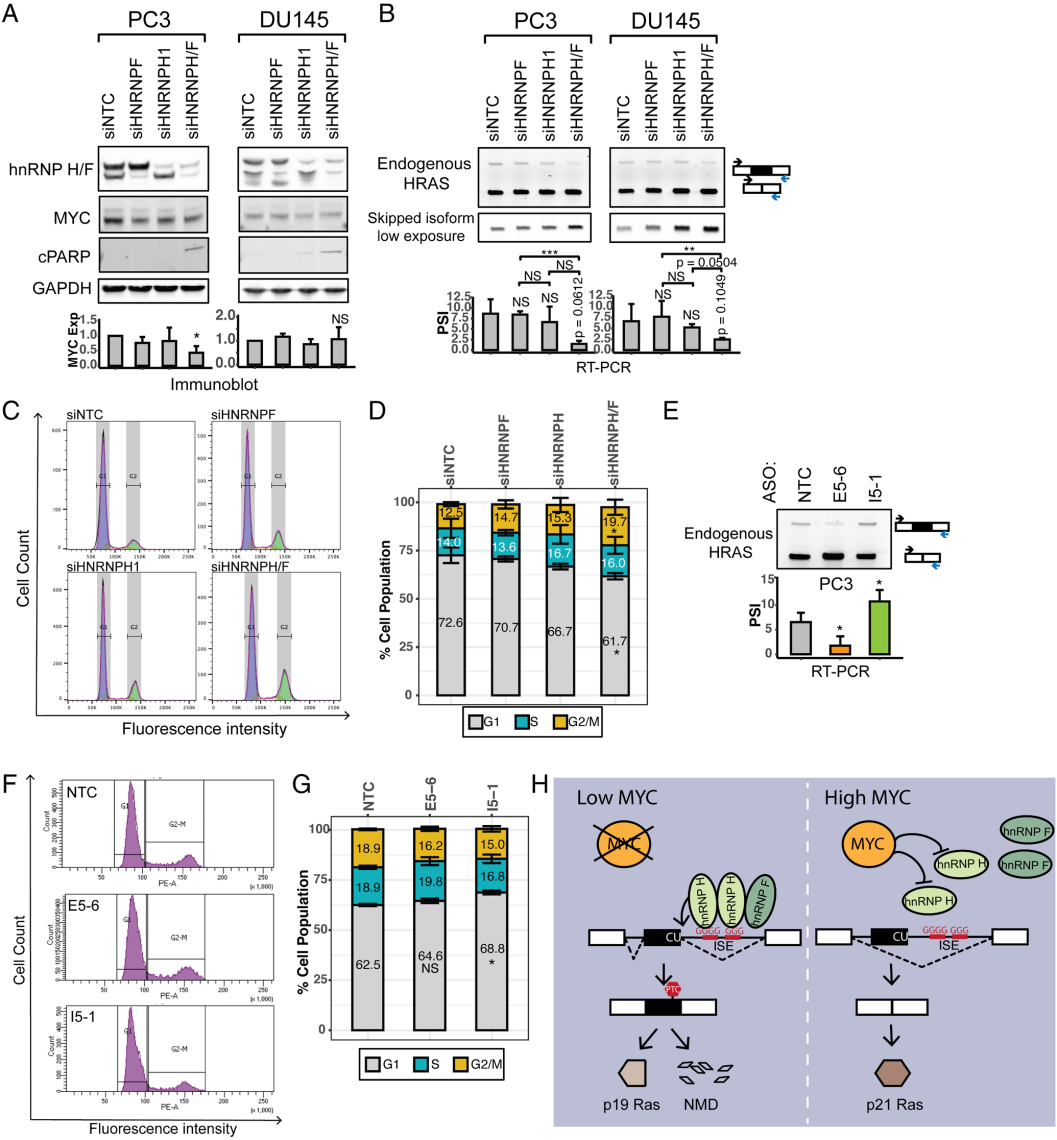
HnRNPs H and F are required for cell proliferation in prostate cancer cell lines. (*A*) The effects of hnRNP H and hnRNP F knockdown on the expression of MYC and the apoptosis marker cPARP in the prostate cancer cell lines, PC3 and DU145. Immunoblot showing the expression levels of hnRNPs H and F, MYC, and cPARP in cells transfected with control, HNRNPF, HNRNPH1, or HNRNPH/F siRNAs. GAPDH was used as a loading control. The bar graphs present the quantification of MYC expression in response to each siRNA perturbation, with the mean +/− SD of triplicates. (*B*) RT-PCR analyses showing the splicing changes of endogenous HRAS transcripts in response to each siRNA perturbation. The bar graphs show the quantification of HRAS exon 5 PSI, with the mean +/− SD of triplicates. (*C*) Cell cycle analysis by FACS of PC3 cells transfected with control, HNRNPF, HNRNPH1, or HNRNPH/F siRNAs, and stained with propidium iodide. (*D*) Stacked bar plot showing the quantification of cells in the G1, S, and G2/M phases. Bars present the mean +/− SD of triplicates. (*E*) RT-PCR analysis showing the splicing changes of endogenous HRAS transcripts in response to each ASO perturbation. The bar graph presents the quantification of RT-PCR results, with the mean +/− SD of triplicates. (*F*) Cell cycle analysis by FACS of PC3 cells transfected with nontargeting control, E5-6 and I5-1 ASOs, and stained with propidium iodide. (*G*) Stacked bar plot showing the quantification of cells in G1, S, and G2/M phases. Bars present the mean +/− SD of triplicates. NS: *P* > = 0.05; *P < 0.05; **P < 0.01; ***P < 0.001 (Student’s *t*-test). (*H*) Model of hnRNP H regulation of HRAS exon alternative splicing under low MYC versus high MYC conditions.

To evaluate the effects of splicing factors hnRNP H and F on the growth of MYC-transformed cells, we knocked down their expression in two prostate cancer cell lines. PC3 is an advanced adenocarcinoma cell line with high metastatic potential, while DU145 cells derive from a prostate carcinoma with moderate metastatic potential (66). PC3 and DU145 cells transfected with siRNAs targeting hnRNPs F, H, or both showed >75% depletion of each factor. The combined depletion of hnRNP H and F induced HRAS exon 5 skipping as seen previously (Fig. 7 *A* and *B*). The knockdown of H or F or both also resulted in a small reduction in MYC in the PC3 cells. The double knockdown also showed an increase in cPARP protein, indicating the induction of cell apoptosis (Fig. 7*A*), and it was apparent that the cultures had stopped proliferating after the hnRNP H/F depletion. To evaluate how loss of hnRNP H and F affected cell growth, we performed flow cytometry of propidium iodide–stained PC3 cells. Depletion of hnRNP F or hnRNP H, and particularly the double knockdown, reduced the number of cells in the G1 phase by 10.9% and increased cells in G2 by 7.2% compared with control cells (Fig. 7 *C* and *D*), indicative of a mitotic block. HnRNPs H and F are thus needed for proper mitotic progression in the prostate cancer cell lines.

To assess the effects of HRAS splicing on prostate cancer cell proliferation, we applied the most active ASOs from our tiling analysis to modulate the HRAS exon 5 inclusion. ASOs E5-6 or I5-1, as well as a nontargeting control, were transfected into PC3 cells. As seen in HEK293 cells, 48 h after transfection, E5-6 reduced exon5 inclusion from 6.5% to 1.7%. In contrast, ASO I5-1 increased exon5 inclusion from 6.5% to 10.7% (Fig. 7*E*). Cell cycle analysis of the ASO-treated PC3 cells indicated that reducing exon 5 splicing with E5-6 had no significant effect on cell cycle progression. However, ASO I5-1 which increased exon 5 splicing led to an increase in the cells in G1 and fewer cells in the S and G2/M phases (Fig. 7 *F* and *G*). This agrees with a previous finding that HRAS p19 overexpression induced a G1/S phase delay in Hela cells (48). Together, our data indicate that modulation of HRAS splicing can impact cell proliferation in prostate cancer cells.

## Discussion

We found that MYC transformation influences the splicing of HRAS proto-oncogene transcripts to decrease exon 5 inclusion and allow greater production of the full-length isoform. We identified clusters of positive and negative splicing regulatory elements in the sequence encompassing exon 5, including splicing enhancers in the intron downstream. We show that these G-run enhancers bind to the splicing regulators hnRNP H and hnRNP F and are required for activating splicing of exon 5. HnRNP H1 expression was found to be anticorrelated with the MYC score across many tumor types including lung, breast, and prostate, consistent with the repression of exon 5 by MYC. HnRNP F showed the opposite correlation. We found that HRAS exon 5 is one of a group of MYC-regulated exons that are also regulated by hnRNPs H and F, which are essential for the growth of prostate cancer cells. These results are summarized in Fig. 7*E*, where the activation of MYC down-regulates hnRNP H, leading to decreased HRAS exon 5 splicing and increased expression of the full-length p21 HRAS oncoprotein.

Human HRAS exon 5 was originally named IDX and shown to carry an in-frame PTC to create a truncated protein (46). Human mutations that reduce IDX inclusion were shown to increase the activity of full-length p21 HRAS (46, 67). HRAS exon 5-containing transcripts were later shown to undergo nonsense-mediated mRNA decay (NMD) in the cytosol, with the undegraded portion found largely in the nucleus (68). We found that cycloheximide treatment does increase this isoform consistent with its loss to NMD (*SI Appendix*, Fig. S7*A*). Thus, one role of the exon seems to be the modulation of p21 HRAS levels and activity.

HRAS transcripts that escape from NMD are potentially translated into the C-terminal-truncated p19 HRAS protein, reported to have a distinct function from the full-length p21 HRAS protein (47, 48). Exon 5 and its flanking introns are conserved in mammals, although the mouse gene does not contain the stop codon and the exon 5-included isoform is predicted to terminate in exon 6. The C-terminal peptides of the truncated isoforms in the human and mouse are quite similar, supporting the idea that p19 is a functional protein variant of HRAS. We have not detected the p19 isoform by immunoblot in our system. However, p19 was detected in the nucleus and cytoplasm of HeLa cells using an antibody specifically targeting its divergent C terminus (47). This p19 form failed to bind to the known p21 interactors Raf1 and Rin1 (47, 48). When overexpressed in different settings, p19 was found to bind a variety of proteins including p73, MDM2, neuron-specific enolase, and RACK and to have varying effects on cell growth and physiology (47, 48, 69–71). These findings indicate that p19 likely serves a separate role from p21 and that modulation of HRAS splicing will alter p19 function in addition to changing p21 activity.

Earlier studies identified a regulatory element called rasISS1 in the intron downstream of HRAS exon 5 that acted to silence exon 5 splicing (58). This inhibition was also observed in an in vitro splicing system, where it required the protein hnRNP A1, and was counteracted by the SR proteins, SRSF2 (SC35) and SRSF5 (SRp40). The RNA-binding proteins FUS/TLS and hnRNP H and the RNA helicase p68 (DDX5) were also found to bind rasISS1. Depletion of p68 in vivo led to increased exon 5 splicing, indicating that it might contribute to splicing repression by rasISS1. The rasISS1 was predicted to form a base-paired stem with exon 5 that may inhibit its splicing, and p68 was shown to unwind the exon 5–rasISS1 stem in vitro. Knockdown of FUS/TLS or hnRNP H reduced the abundance of p19 HRAS protein in vivo (72). These and our findings indicate that HRAS exon 5 is modulated by a combination of positive and negative acting factors as seen with most alternative exons.

The ASO tiling approach allowed us to map regulatory elements more comprehensively across the exon 5 region. ASOs I5-1 and I5-2 target the rasISS1 element, and the activation of splicing by I5-1 indicates the presence of a silencer. Motif-based RBP prediction identified potential hnRNP A1–binding sites within the I5-1 and I5-2-targeted region (*SI Appendix*, Fig. S2*A*), consistent with the previous studies of rasISS1 (58). The limited effect of ASO I5-2 may come from its inability to disrupt the proposed rasISS1 secondary structure. In addition to the rasISS1 silencing activity, our analysis identified enhancer elements near the 3’ end of the ISS and downstream containing G-runs that bind hnRNPs H and F. We find that these proteins strongly activate splicing despite the presence of the rasISS1, and this activation requires the G-runs in the I5-3 region. The ASO tiling also indicated the presence of multiple exonic enhancers within exon 5. Some of these may recruit SRSF2 and SRSF5 whose activity on the exon was previously reported (58). In transient overexpression experiments, we confirmed that SRSF5 activates HRAS exon 5 (*SI Appendix*, Fig. S7*B*). The ASO tiling can be refined, and in future work, we can more precisely delineate the cis-elements using overlapping oligos to identify those that most strongly shift exon 5 splicing and the expression of the p21 HRAS oncoprotein. These can then be tested for effects on tumor growth. Other studies examining the programs of splicing regulation in cancer have also identified HRAS as a MYC-dependent exon. Expression of several SR proteins is altered in response to MYC (73, 74), and it was recently reported that some SR proteins, particularly SRSF2, act to repress HRAS cassette exon splicing in MYC-active tumors (43). This study also mapped cis-regulatory elements of HRAS splicing using CRISPR-guided artificial splicing factors, revealing position-dependent effects of SR proteins on HRAS exon 5.

HnRNP H has been connected to other aspects of Ras signaling. Studies of the A-Raf kinase found that splicing to create its full-length isoform required hnRNP H (33, 34). This isoform inhibits apoptosis in tumor cells through interaction with the MST2 kinase. The short A-Raf isoform expressed in low–hnRNP H conditions can act as a dominant negative protein to suppress Ras activation and oncogenic transformation. These studies found that high MYC correlated with high hnRNP H expression in HeLa and several other tumor cell lines, the opposite of the correlation we observe in most tumors in the TGCA database. It will be interesting to investigate whether these different results arise from differences between cell lines and primary tumors, differences between tumor types, or some other differences between the systems. Another earlier study found that hnRNP F affected cell proliferation through interactions with mTOR and the S6 kinase 2 pathway (75). We found that both hnRNP H and F are required for growth of a prostate cancer cell line. It will be interesting to assess the signaling pathways involved in these effects on cell proliferation control and whether the Ras-MEK-ERK or Ras-PI3K-AKT pathways are involved.

There are several findings within our data that remain unexplained. One question is the apparent upregulation of hnRNP F by MYC. Since hnRNP F also seems to stimulate HRAS exon 5, one would expect it to go down with increased MYC. However, the cross-regulation by the hnRNP H and F paralogs (Fig. 3*A*) makes it challenging to disentangle their individual contributions. Since we observe hnRNP F to increase in many tumors, where hnRNP H is decreased, it is possible that one role of hnRNP F is to down-regulate hnRNP H, which is the stronger activator of HRAS exon 5 splicing (Fig. 5*A* and *SI Appendix*, Fig. S4*A*). It is possible that other factors, of the many affecting exon 5, are also counteracting the effect of hnRNP F. It should also be noted that MYC correlations with gene expression are only measuring RNA, and it is possible the proteins encoded by these mRNAs are behaving differently. It is also possible that modifications of the hnRNP F protein could alter its activity. Another question regards the requirement for hnRNP H in the growth of MYC-transformed cells. Although reduced splicing of HRAS exon 5 resulting from reduced hnRNP H is apparently conducive to growth, some level of hnRNP H is still required. What mRNA isoforms are responsible for this hnRNP H dependence will be interesting to investigate.

## Materials and Methods

Descriptions of minigene reporters and cDNA expression vectors, cell culture, plasmid and siRNA transfections, RNA isolation, RT-PCR and RT-qPCR, immunoblotting, cell cycle analysis, RNA-seq data processing, gene expression and splicing analysis for cell lines, and pan-cancer analysis are available in *SI Appendix*.

### ASO Transfections.

ASOs were provided by IONIS Pharmaceuticals and have uniform phosphorothioate backbone chemistry with modified 2′ methoxyethyl sugars. HEK293 or PC3 cells were plated the day before transfection in 12-well culture plates. ASOs (100 nM) were transfected into cells using Lipofectamine 2000 (Invitrogen). HEK239 cells were harvested 24 h after transfection for RT-PCR analyses. PC3 cells were harvested 48 h after transfection for RT-PCR and cell cycle analysis. A list of ASO sequences is presented in *SI Appendix*, Table S1.

### RNA Immunoprecipitation.

Antibodies targeting proteins of interest or IgG isotype control (5 μg) were incubated with 20 μL Dynabeads Protein G (Thermo Fisher Scientific) in buffer WB150 (20 mM HEPES-KOH, pH 7.5, 150 mM NaCl, and 0.1% Triton-X100) at 4 °C for 2 h. HEK293 cells were harvested and sonicated in cold lysis buffer (20 mM HEPES-KOH, pH 7.5, 150 mM NaCl, 0.5% Triton X-100, 0.05% SDS, 1 mM EDTA, 0.5 mM DTT, 1× protease inhibitors, and 100 U/mL RNaseOUT). After centrifugation at 20,000 *g* for 10 min at 4 °C, the supernatant was incubated with antibody-conjugated beads at 4 °C for 3 h. The beads were washed five times with buffer WB150, and RNA was extracted with TRIzol.

## Supplementary Material

Appendix 01 (PDF)

Dataset S01 (XLSX)

## Data Availability

All study data are included in the article and/or *supporting information*. Previously published data were used for this work (GSE141633 (42); TCGA https://www.cancer.gov/tcga (52); GTEx https://www.commonfund.nih.gov/gtex (51); ENCODE https://www.encodeproject.org/ (63)).
