# Supplementary material for: The RNA-binding proteins hnRNP H and F regulate splicing of a MYC-dependent HRAS exon in prostate cancer cells
Source: Proc Natl Acad Sci U S A. 2023 Jul 3;120(28):e2220190120. doi: 10.1073/pnas.2220190120 (PMC10334793; doi:10.1073/pnas.2220190120)
Supplement: Supplementary file 1 — Appendix 01 (PDF) [file pnas.2220190120.sapp.pdf]

PNAS

Supporting Information for

The RNA binding proteins hnRNP H and F regulate splicing of a MYC-dependent HRAS exon in Prostate Cancer Cells

Xinyuan Chen[1], Harry Taegyun Yang[2], Beatrice Zhang[3], John W. Phillips[4], Donghui Cheng[4], Frank Rigo[5], Owen N. Witte[4,6,7,8,9], Yi Xing[3,10,*], Douglas L. Black[4,7,8,9,*]

[1]Molecular Biology Interdepartmental Doctoral Program, University of California, Los Angeles, Los Angeles, California 90095, USA.
[2]Bioinformatics Interdepartmental Graduate Program, University of California, Los Angeles, California 90095, USA.
[3]Center for Computational and Genomic Medicine, The Children's Hospital of Philadelphia, Philadelphia, Pennsylvania 19104, USA.
[4]Department of Microbiology, Immunology, and Molecular Genetics, University of California, Los Angeles, Los Angeles, California 90095, USA.
[5]Ionis Pharmaceuticals, Inc., 2855 Gazelle Ct., Carlsbad, California 92010, USA.
[6]Department of Molecular and Medical Pharmacology, University of California, Los Angeles, California 90095, USA.
[7]Jonsson Comprehensive Cancer Center, University of California, Los Angeles, California 90095, USA.
[8]Eli and Edythe Broad Center of Regenerative Medicine and Stem Cell Research, University of California, Los Angeles, California 90095, USA.
[9]Molecular Biology Institute, University of California, Los Angeles, California 90095, USA.
[10]Department of Pathology and Laboratory Medicine, University of Pennsylvania, Philadelphia, Pennsylvania 19104, USA.

*Douglas L. Black, Yi Xing
Email: dougb@microbio.ucla.edu; xingyi@chop.edu

This PDF file includes:

SI Materials and Methods
Figures S1 to S7
Tables S1 to S4
SI References

Other supporting materials for this manuscript include the following:

Dataset S1

SI Materials and Methods

Construction of minigene reporters and cDNA expression vectors

The HRAS minigene reporter was constructed by PCR amplifying a ~900bp region spanning exon 4 to exon 6 from human genomic DNA isolated from HEK293 cells using Phusion high-fidelity DNA polymerase (NEB). The PCR fragment were inserted into pcDNA3.1(+) vector through restriction sites BamHI/EcoRI. G-run point mutations were introduced using site-directed mutagenesis. Construction of the C-terminal 6xHistidine tagged hnRNP H and hnRNP F cDNA constructs, and siRNA-resistant hnRNP H construct was described previously (1). Mutations to create the siRNA-resistant hnRNP F construct were introduced by site-directed mutagenesis. hnRNP H coding sequences were subcloned into C-terminal p3xFLAG CMV vector. All the constructs were confirmed by sequencing.

Cell culture, plasmid and siRNA transfections

HEK293, HepG2, PC3, DU145 cells were maintained in DMEM, EMEM, F12K, RPMI1640, respectively, all supplemented with 10% fetal bovine serum at 37C in 5% CO_2. Cells were plated the day before transfection in a 6-well culture plate. Plasmids (3ug per well) were transfected into HEK293 cells using lipofectamine 2000 (Invitrogen), according to manufacturer's instructions. Cells were harvested 48hrs post transfection for RT-PCR and immunoblotting analyses. siRNAs were transfected into HEK293 (10nM) or HepG2, PC3 and DU145 (20nM) using lipofectamine RNAiMAX (Invitrogen), according to manufacturer's instructions. 24hrs after transfection, siRNAs were transfected a second time. 72hrs after the first siRNA transfection, cells were harvested for RT-PCR and immunoblotting analyses. A list of siRNA sequences is presented in the supplemental table S3.

RNA isolation, RT-PCR, RT-qPCR

Total RNA was isolated using TRIzol Reagent (Life Technologies), followed by DNase I treatment (Invitrogen Ambion), and then extracted again with acid phenol chloroform (Invitrogen Ambion). 500ng – 1ug of total RNA was subjected to cDNA synthesis using SuperScript III reverse transcriptase (Thermo Fisher Scientific), primed by random hexamers. PCR was conducted using GoTaq, 18 cycles for minigene reporters and 24 cycles for endogenous RNAs. PCR products were run on 5% PAGE, stained with SYBRGold (Thermo Fisher Scientific) (unless otherwise specified), and scanned with a Typhoon imager (GE healthcare). PCR band intensities were quantified using ImageJ. RT-qPCR was conducted with SensiFast SYBR lo-ROX Kit (Bioline) on a Quant Studio 6 Flex real-time PCR system (Thermo Fisher Scientific). RT-PCR primers are listed in the supplemental table S2.

Immunoblotting

Proteins were extracted using RIPA lysis buffer (Thermo Fisher Scientific) supplemented with Benzonase Nuclease (Sigma Aldrich) and protease inhibitor cocktail (Sigma Aldrich). Protein concentrations were quantified by BCA protein assay kit (Pierce Biotechnology). Cell lysates were prepared with SDS loading buffer (50 mM Tris·Cl, pH 6.8, 0.05% bromophenol blue, 10% glycerol, 2% SDS, and 0.1 M DTT) and proteins were separated on SDS-PAGE gels and transferred to PVDF membranes. Antibodies used in this study were listed in the supplemental table S4. Western blot images were scanned on a Typhoon imager (GE healthcare) and band intensities were quantified using ImageJ.

Cell cycle analysis

PC3 cells were plated the day before transfection in a 6-well or 12-well culture plate. siRNA or ASO were transfected as described above. 72hrs after siRNA transfection or 48hrs after ASO transfection, cells were trypsinyzed and washed with PBS. These cell suspensions were fixed with 70% ice-cold ethanol overnight at -20C. Cells were stained with propidium iodide at 75 ug/ml and incubated at 37C for 30min, followed by FACS analysis.

RNA-seq data processing, gene expression and splicing analysis for cell lines

The fastq files of RBP CRISPR RNA-seq and shRNA RNA-seq in HepG2 and K562 cells were downloaded from ENCODE Consortium (2) (https://www.encodeproject.org/) (HepG2 HNRNPF CRISPR KO, ENCODE accession no.: ENCSR599NNK; HepG2 HNRNPH1 CRISPR KO: ENCSR094HEU; K562 HNRNPF shRNA KD: ENCSR392HSJ; K562 HNRNPH1 CRISPR KO: ENCSR354RSR; HepG2 SRSF4 KO: ENCSR929JKA; HepG2 SRSF7 KO: ENCSR845MNM; HepG2 SRSF9 KO: ENCSR814JQP; HepG2 SF3A1 KO: ENCSR556TJU; HepG2 RBM5 KO: ENCSR606PVX; HepG2 RBM14 KO: ENCSR166MWM; HepG2 RBM19 KO: ENCSR532IHF;HepG2 RBM25 KO: ENCSR739ZMF). The RNA-seq fastq files of Myc cell lines were downloaded from Gene Expression Omnibus (accession no. GSE141633) via fastq-dump in SRA toolkit. The raw sequencing reads were aligned to human reference genome and gene annotation (GRCh37, GENCODE release 26) using STAR (v2.7.3a) (3). Alternative splicing in the samples was quantified as Percent Spliced In (PSI, ψ) using rMATS-turbo (v4.1.1) (4) by using the same gene annotation as the alignment step. Skipped exon (SE) events with insufficient average coverage, $IC + SC \leq 10$, were excluded from further analysis. Significant SE events were identified using $|\Delta\psi| > 0.05\ and\ FDR < 0.05$, calculated using PAIRADISE (5) with the equal variance option. Gene Ontology analysis was done using PANTHER (6), p-value was computed using Fisher's exact test, and the Benjamini-Hochberg procedure was used to control for FDR < 0.05.

RNA-seq data compilation and processing for the pan-cancer analysis

RNA-seq data of tissue samples were compiled from two public domains: TCGA (7), GTEx (8). Tumor tissue samples were obtained from TCGA while normal tissue was obtained from GTEx. The TCGA RNA-seq fastq files were downloaded from GDC through their GDC Data Transfer Tool Client (9) while the GTEx files were downloaded from dbGAP (10) through fastq-dump from the SRA toolkit. The TCGA and GTEx samples were matched by tissue origin (11). The fastq files were mapped by STAR 2.5.3a (3). The STAR 2-pass function was enabled to improve the accuracy of the alignment. The genome annotation file was obtained from GENCODE V26 (12) under human genome version hg19. Gene expression quantification and alternative splicing quantification were done using Cufflinks v2.2.1 (13) and rMATS 4.1.0 (4) respectively. Percent Spliced In (PSI) ratio was used as a statistic to quantify alternative splicing events from the rMATS output. Splicing events were filtered for splice junction reads >= 10 (otherwise the sample's PSI value is marked with 'NA'), PSI range (i.e. difference between maximum and minimum PSI values) > 5%, and for mean skipping or inclusion values over 5% across the entire dataset, with fewer than 50% of samples missing values.

Pathway Enrichment-Guided Activity Study of Alternative Splicing for the pan-cancer analysis

The PEGASAS pipeline (14) was used to calculate pathway activity scores and identify alternative splicing events that correlate with the signaling pathway of interest. The Myc Targets V2 hallmark gene signature list obtained from The Molecular Signatures Database (MSigDB) (15) was used for pathway activity score calculation. Myc pathway scores were calculated using cufflinks expression outputs. Alternative splicing events (SE) correlated with the Myc signaling pathway were identified through the PEGASAS correlation step. A correlation permutation test was used to acquire empirical p-values, by permuting the pathway scores 5,000 times. Highly correlated events were defined as events with an empirical p-value < 0.0002 and with a |Pearson correlation coefficient| > 0.3, as in previous study (14).

Gene expression values for RBP correlation

FeatureCounts v2.0.1 was used to quantify reads from aligned BAM files (16). DESeq2 v1.26.0 was used to normalize gene expression (17). The normalized gene expression values for HNRNPH1 and HNRNPF along with 218 other splicing factors were correlated with Myc scores (18).

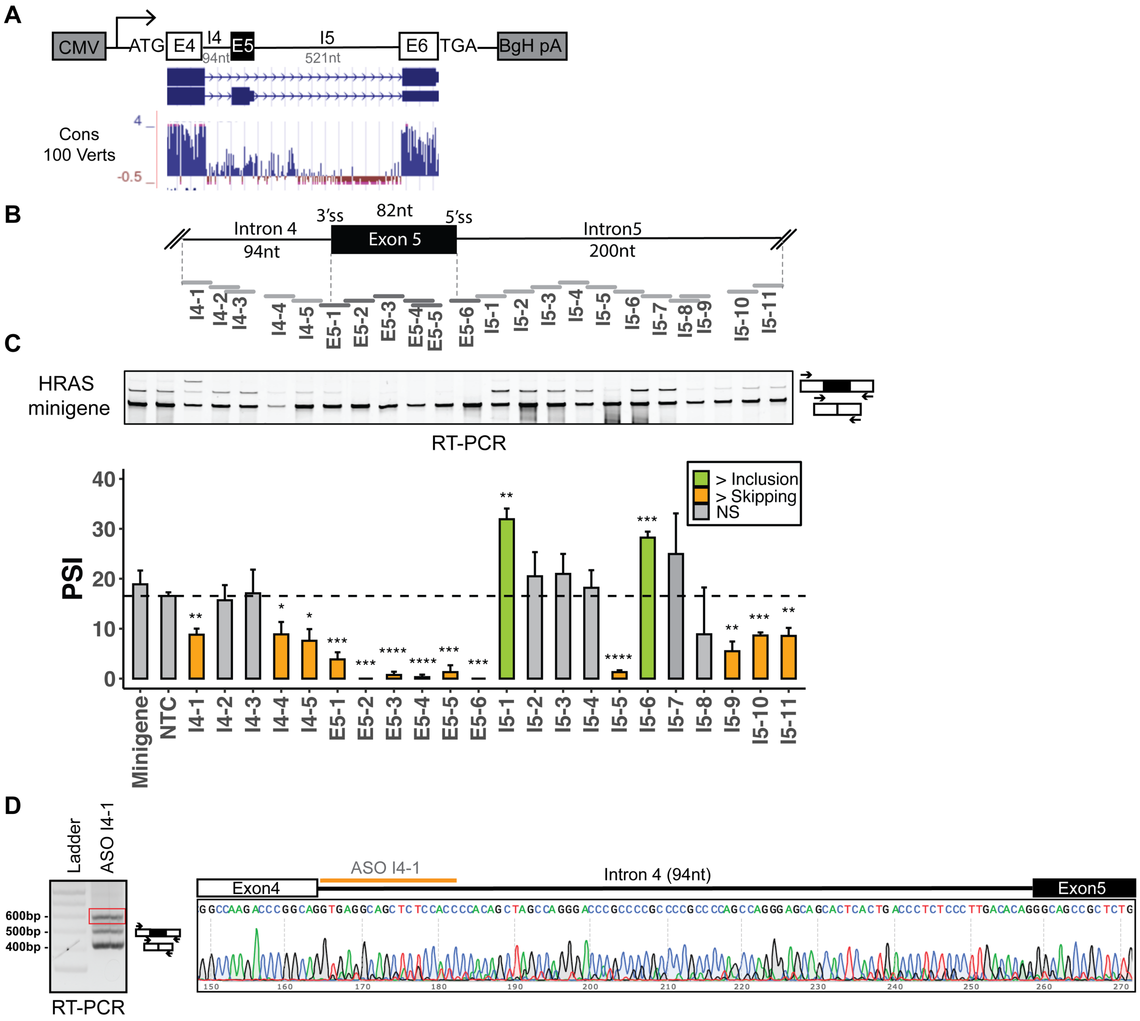

Fig. S1. Identification of splicing cis-regulatory elements with HRAS minigene reporter.

(A) Diagram of HRAS minigene reporter with a schematic representation of gene structure and conservation. **(B)** Schematic of ASO tiling across HRAS cassette exon and its partial flanking introns on minigene reporter. A 376-nucleotide region tiled by non-overlapping 18-mer ASOs. Each horizontal bar represents an ASO. **(C)** Semi-quantitative RT-PCR analyses showing the effects of ASOs on HRAS minigene reporter. The arrows indicate the locations of minigene-specific primers. The bar graph shows the quantification of RT-PCR results calculated as percent-spliced-in (PSI) (gray: control or no significant changes; orange: more skipping; green: more inclusion). Each bar shows the mean PSI +/- SD of three biological replicates. NTC, non-targeting control. Minigene, no-ASO mock control. NS: p > = 0.05; *: p < 0.05;**: p < 0.01; ***: p < 0.001; ****: p < 0.0001 (Student's t-test). **(D)** RT-PCR gel showing the cryptic band (in red rectangle) in minigene reporter, induced by ASO I4-1 transfection (left). Diagram showing the sequencing result of the cryptic band (right).

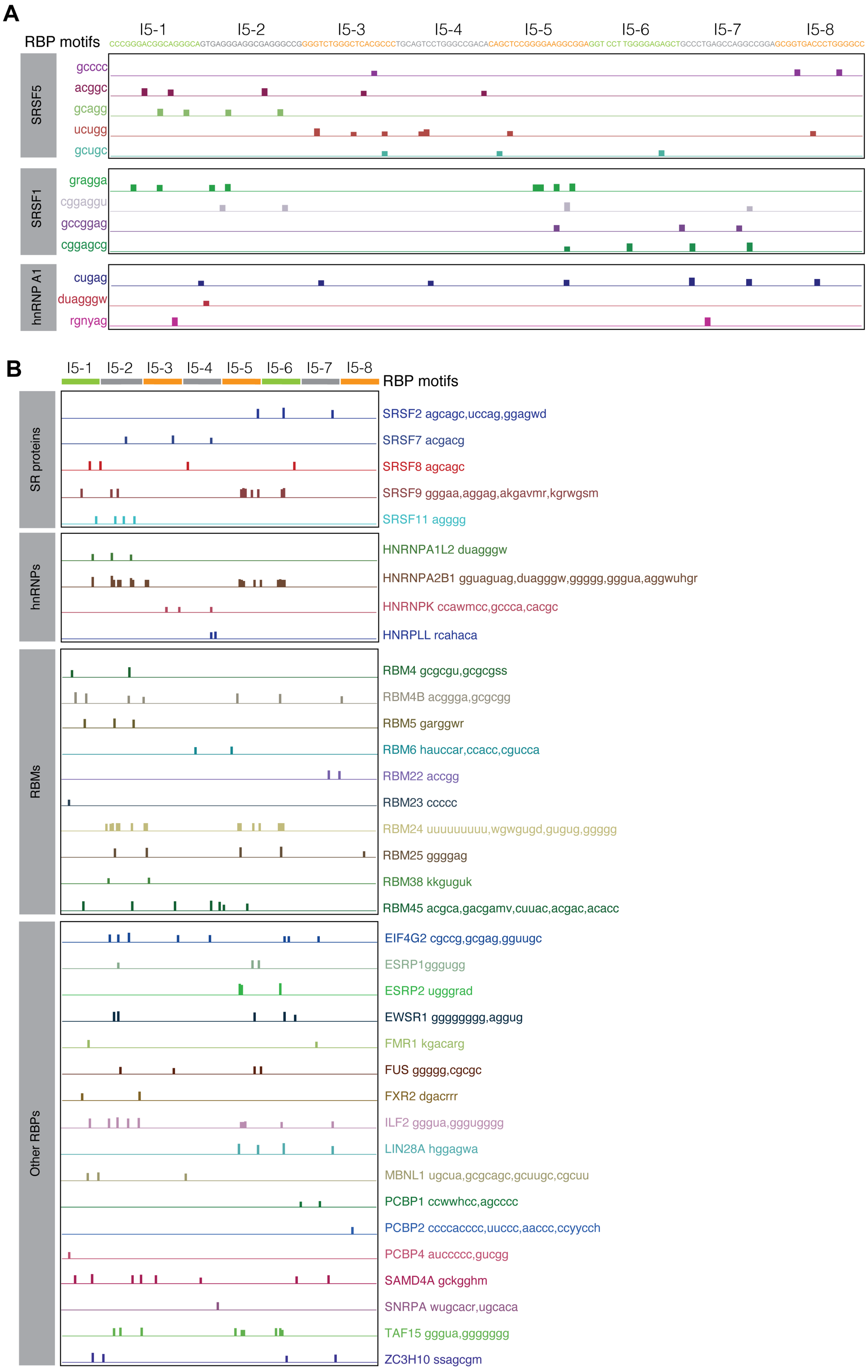

A
RBP motifs
I5-1
I5-2
I5-3
I5-4
I5-5
I5-6
I5-7
I5-8
CCCGGGACGGCAGGGCAGTGAGGGAGGCGAGGGCCGGGGTCTGGGCTCACGCCCTGCAGTCCTGGGCCGACACAGCTCCGGGGAAGGCGGAGGT CCT TGGGGAGAGCTGCCCTGAGCCAGGCCGGAGCGGTGACCCTGGGGCC
SRSF5
gcccc
acggc
gcagg
ucugg
gcugc
SRSF1
gragga
cggaggu
gccggag
cggagcg
hnRNP A1
cugag
duagggw
rgnyag
B
I5-1
I5-2
I5-3
I5-4
I5-5
I5-6
I5-7
I5-8
RBP motifs
SR proteins
SRSF2 agcagc,uccag,ggagwd
SRSF7 acgacg
SRSF8 agcagc
SRSF9 gggaa,aggag,akgavmr,kgrwgsm
SRSF11 agggg
hnRNPs
HNRNPA1L2 duagggw
HNRNPA2B1 gguaguag,duagggw,ggggg,gggua,aggwuhgr
HNRNPK ccawmcc,gccca,cacgc
HNRPLL rcahaca
RBMs
RBM4 gcgcgu,gcgcgss
RBM4B acggga,gcgcgg
RBM5 garggwr
RBM6 hauccar,ccacc,cgucca
RBM22 accgg
RBM23 ccccc
RBM24 uuuuuuuuu,wgwgugd,gugug,ggggg
RBM25 ggggag
RBM38 kkguguk
RBM45 acgca,gacgamv,cuuac,acgac,acacc
Other RBPs
EIF4G2 cgccg,gcgag,gguugc
ESRP1gggugg
ESRP2 ugggrad
EWSR1 gggggggg,aggug
FMR1 kgacarg
FUS ggggg,cgcgc
FXR2 dgacrrr
ILF2 gggua,gggugggg
LIN28A hggagwa
MBNL1 ugcua,gcgcagc,gcuugc,cgcuu
PCBP1 ccwwhcc,agcccc
PCBP2 ccccacccc,uuccc,aaccc,ccyych
PCBP4 auccccc,gucgg
SAMD4A gckgghm
SNRPA wugcacr,ugcaca
TAF15 gggua,ggggggg
ZC3H10 ssagcgm

Fig. S2. RBPmap identifies binding motifs of potential splicing regulators in the HRAS intron 5 sequence.

(A) UCSC genome browser visualization of SpliceAid2 and RBPmap-predicted binding sites of SRSF5, SRSF1, and hnRNP A1 on HRAS intron 5. Binding motifs for each splicing factor were compiled RBPmap and SpliceAid2 database (19, 20). The sequences targeted by each ASO are highlighted in orange (more skipping), green (more inclusion), gray (no significant change). **(B)** USCS genome browser tracks showing binding motifs of additional factors identified by RBPmap, including SR proteins, hnRNPs, RBMs, and others.

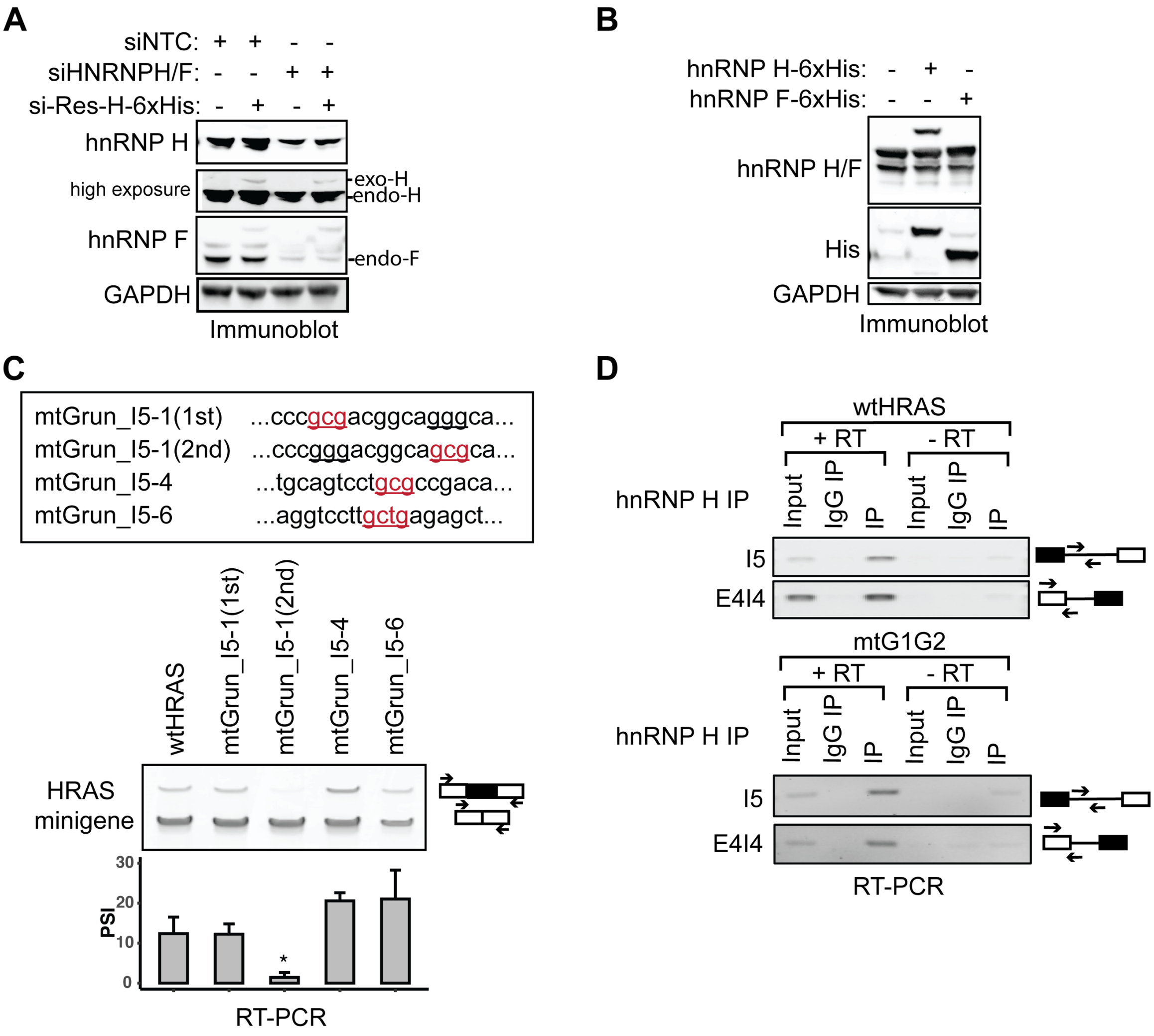

Fig. S3. Rescue of hnRNP H expression after hnRNPs H and F knockdown in HEK293 cells; Splicing analyses of additional Grun mutations; Immunoprecipitated RNA is free of minigene DNA contamination.

(A) Separate blots of hnRNP H and hnRNP F using antibodies specific to the hnRNP H or hnRNP F (Related to Figure 3C). HnRNP H-specific antibody does not react as well with the recombinant hnRNP H. **(B)** Immunoblot showing the expression of endogenous hnRNP's H and F and His-tagged ectopic proteins. GAPDH was used as the loading control. **(C)** Diagram showing additional Gruns in each ASO targeted region, with the mutated sequences underlined and labeled in red. RT-PCR analyses showing the splicing changes of wild type and mutant minigene reporters, wt, wild type; mt, mutant. The bar graph presents the quantification of RT-PCR results, with the mean +/- SD of three biological replicates. *: $p < 0.05$ (Student's t-test). **(D)** RT-PCR of anti-hnRNP H antibody immunoprecipitated RNA using primer pairs amplifying a segment of intron5 or the exon4-intron4 junction, in the presence [+] or absence [-] of reverse transcriptase.

Immunoprecipitated RNA (80%) was amplified with 5 more PCR cycles than the Input control (5%). wt, wild type; mt, mutant.

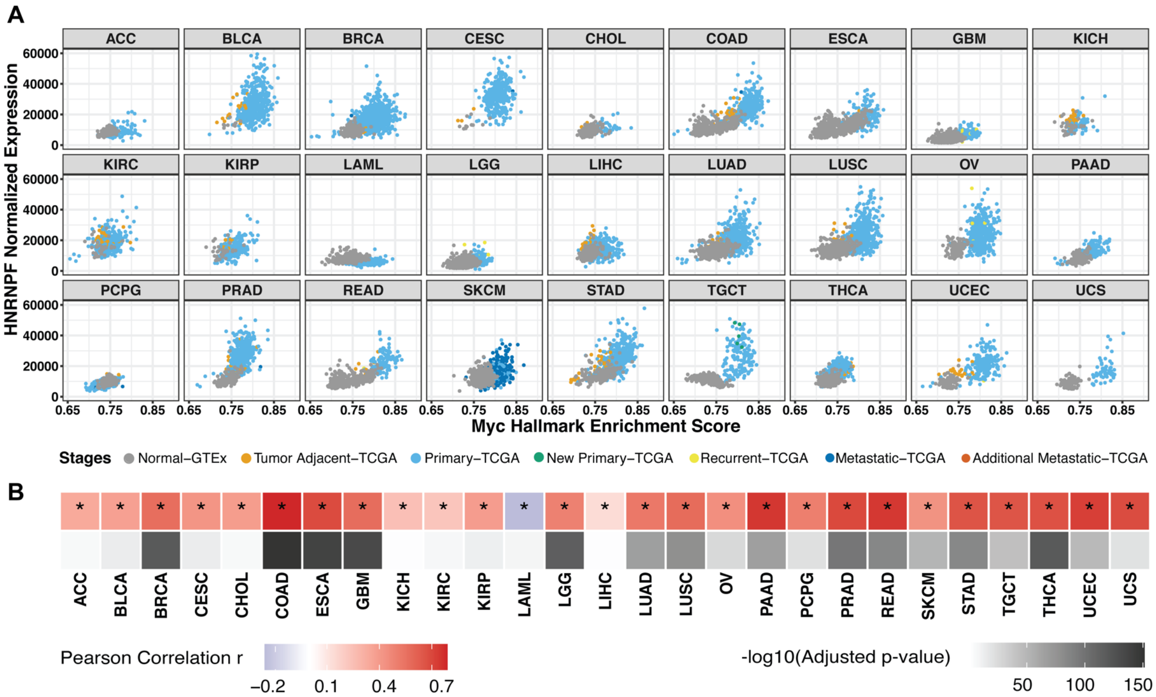

Fig. S4. HNRNPF gene expression increases with Myc activation across tumor types.

(A) Scatterplot matrix showing the correlation of normalized HNRNPF RNA level versus Myc hallmark enrichment score spanning the disease spectrum in multiple tumor types. **(B)** Heatmap summarizing the Pearson correlation coefficient for each tumor type, accompanied with its corresponding adjusted p-value. *, tumor types with statistically significant adjusted p-value (< 0.05).

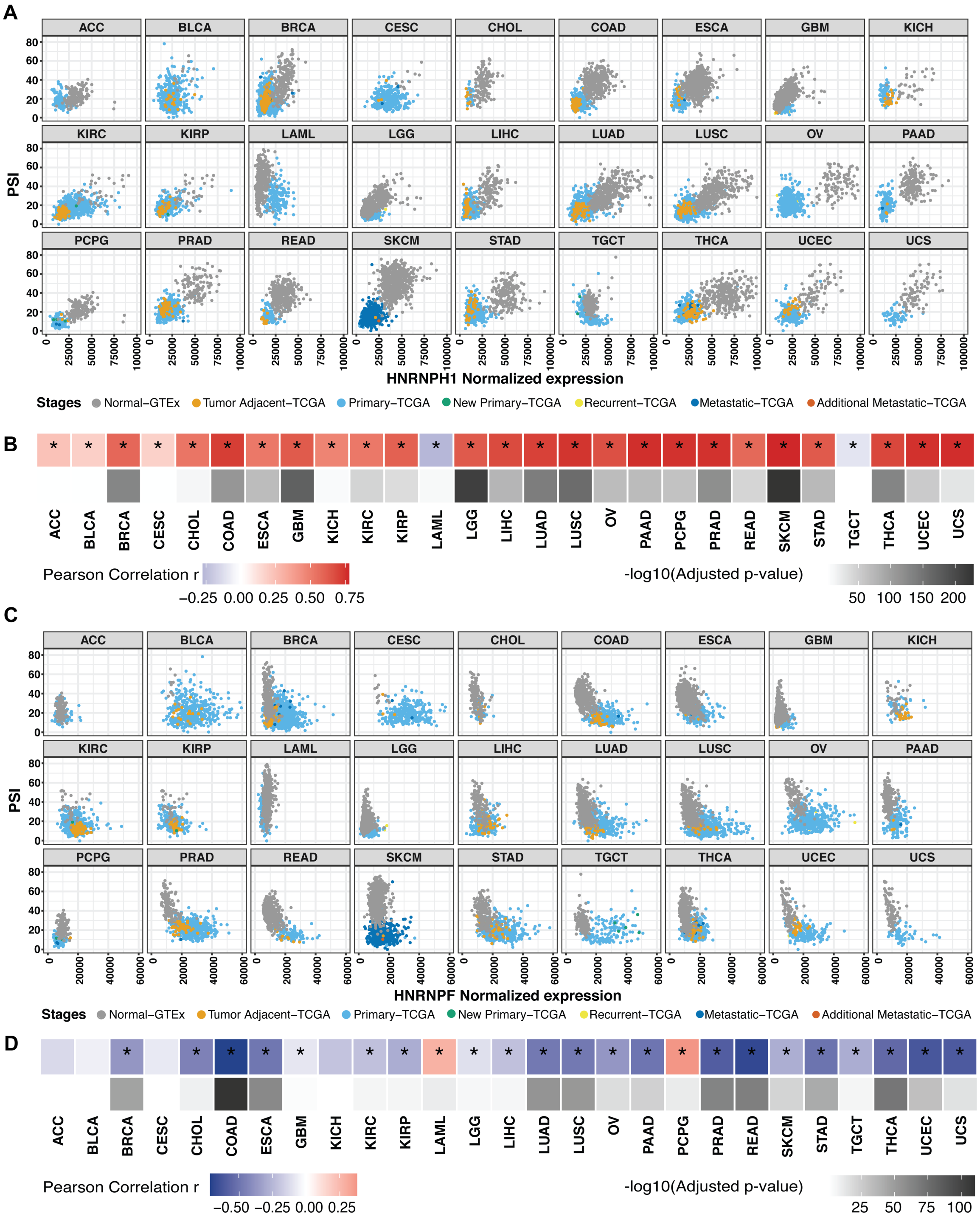

Fig. S5. HRAS exon 5 splicing increases with HNRNPH1 expression but decreases with HNRNPF expression across tumor types.

(A) Scatterplot matrix showing the correlation of HRAS exon 5 inclusion (PSI) with normalized HNRNPH1 RNA level across the disease spectrum of multiple tumor types. **(B)** Heatmap

summarizing the Pearson correlation coefficient between HRAS PSI and HNRNPH1 normalized expression for each tumor type, accompanied with its corresponding adjusted p-value. *, tumor types with statistically significant adjusted p-value (< 0.05). **(C)** Scatterplot matrix showing the correlation of HRAS exon 5 inclusion with normalized HNRNPF RNA level spanning the disease spectrum of multiple tumor types. **(D)** Heatmap summarizing the Pearson correlation coefficient between HRAS PSI and HNRNPF normalized expression for each tumor type, accompanied with its corresponding adjusted p-value. *, tumor types with statistically significant adjusted p-value (< 0.05).

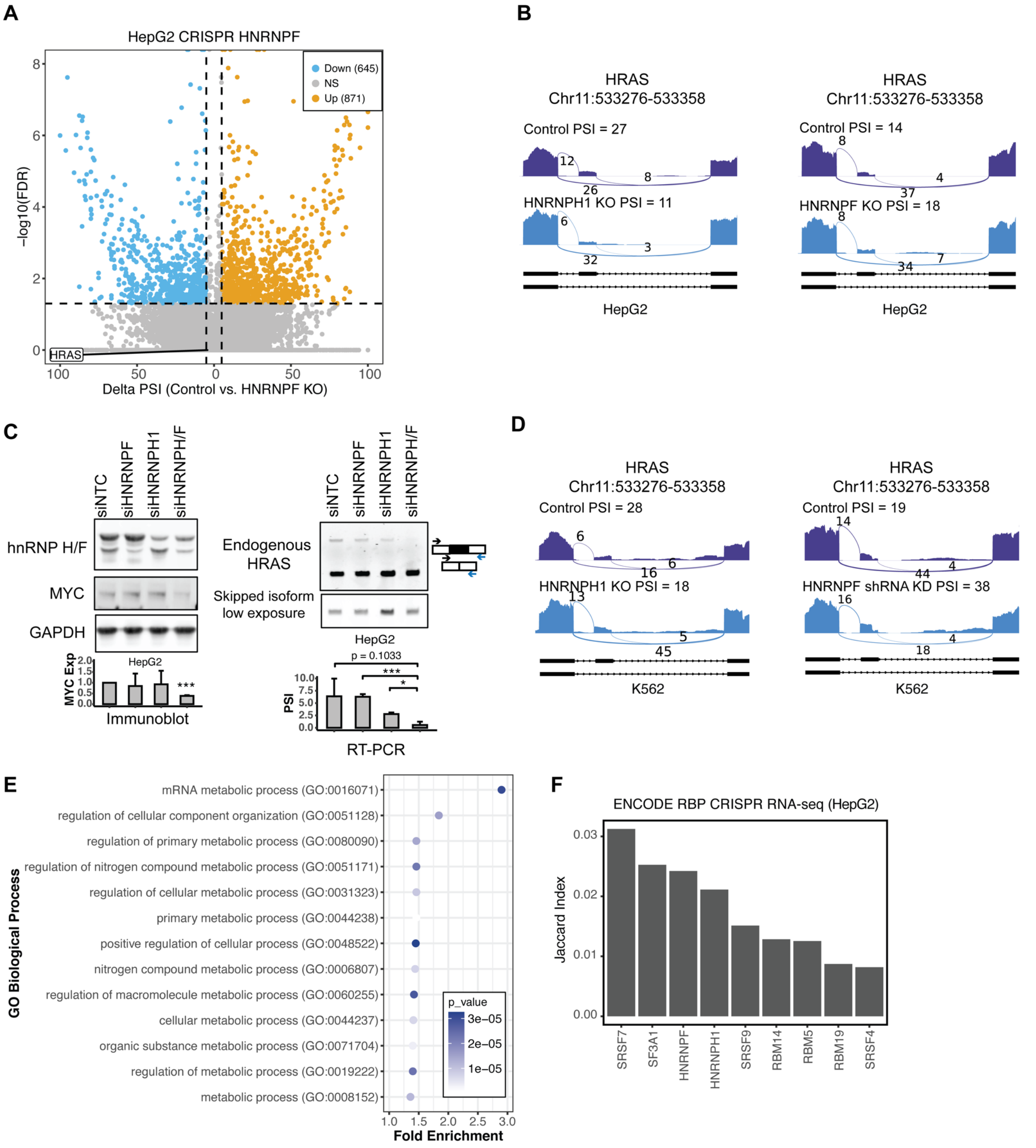

Fig. S6. Differential splicing analysis after HNRNPH/F or other RBP depletion from HepG2 and K562 cells.

(A) Scatterplot showing the skipped exon (SE) events detected after HNRNPF CRISPR knockout in HepG2 cells compared to control. RNA-seq data from ENCODE was analyzed. Significant SE events were filtered by junction reads per event > 10, |deltaPSI| > 0.05, and FDR < 0.05. NS, non-significant. **(B)** Sashimi plot showing the PSI of HRAS exon 5 in HNRNP H1 KO (left) or HNRNPF KO (right) compared with control in HepG2 cells. KO, knockout. **(C)** The effects of hnRNP H/F knockdown on HRAS splicing in HepG2 cells. Immunoblot showing the protein levels

of hnRNP H/F and MYC in HepG2 cells after transfection with control, HNRNPF, HNRNPH1, or HNRNPH/F siRNAs. GAPDH was used as a loading control. The bar graph (left) shows the quantification of MYC expression in response to each siRNA perturbation, with the mean +/- SD of three biological replicates. RT-PCR analysis of the splicing of endogenous HRAS transcripts after siRNA treatment. The bar graph (right) shows the quantification of HRAS PSI, with the mean +/- SD of three biological replicates. *: $p < 0.05$; ***, $p < 0.0001$ (Student's t-test). **(D)** Sashimi plots showing the PSI of HRAS exon 5 in HNRNPH1 KO **(left)** or HNRNPF KD **(right)** compared with control in K562 cells. KD, knockdown. **(E)** Gene ontology analyses using PANTHER for genes represented by SE events between MYC and HNRNPH, between MYC and HNRNPF, or between both comparisons. **(F)** Bar plot of the Jaccard index (Overlap/Union) measuring the overlap of MYC-dependent SE events and RBP-dependent SE events. CRISPR KO RNA-seq data in HepG2 cells from ENCODE was analyzed. Significant SE events were filtered by junction reads per event > 10, |deltaPSI| > 0.05, and FDR < 0.05. Jaccard index was calculated by shared SE events over all SE events for each pairwise comparison.

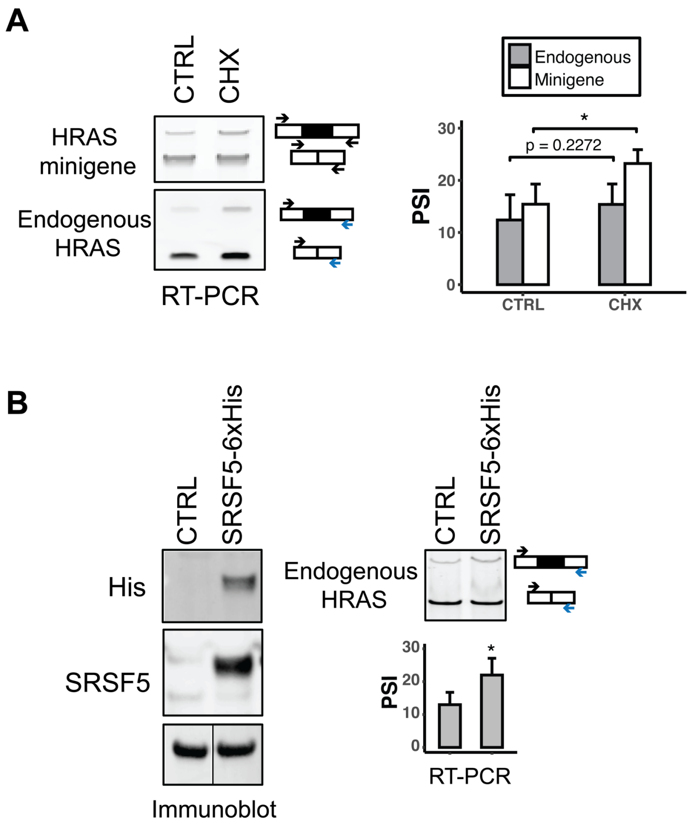

Fig. S7. HRAS exon 5 included transcript is a NMD target; SRSF5 activates HRAS exon 5 inclusion.

(A) The effect of cycloheximide treatment on HRAS exon5-containing transcripts in HEK293 cells. Cells were treated with 100ug/ml cycloheximide (Sigma Aldrich C1988) for 2hrs before lysis. RT-PCR analysis of the splicing of HRAS minigene and endogenous transcripts after cycloheximide treatment. CTRL, control; CHX, cycloheximide. The bar graph shows the quantification of HRAS PSI, with the mean +/- SD of three biological replicates. *: $p < 0.05$ (Student's t-test). **(B)** The effects of SRSF5 overexpression on HRAS splicing in HEK293 cells. Immunoblot showing the protein level of His-tagged SRSF5. RT-PCR analysis of the splicing of endogenous HRAS transcripts after transient SRSF5 overexpression. The bar graph shows the quantification of HRAS PSI, with the mean +/- SD of three biological replicates. *: $p < 0.05$ (Student's t-test).

Table S1. List of ASOs

Name	Sequence 5'-3'
NTC	TCTATTAGCTAACAAATA
I4-1	GGTGGAGAGCTGCCTCAC
I4-2	GTCCCTGGCTAGCTGTGG
I4-3	GGGCGGGTCCCTGGCTAG
I4-4	TGCTGCTCCCTGGCTGGG
I4-5	AGGGAGAGGGTCAGTGAG
E5-1	AGCGGCTGCCCTGTGTCA
E5-2	AGCTGGAGCTAGAGCCAG
E5-3	GGTCCCAGAGGGTCCCGG
E5-4	GGTCACATGGGTCCCGGG
E5-5	GGGTCACATGGGTCCCGG
E5-6	AGACTTACAGCGCGAGGG
I5-1	CTGCCCTGCCGTCCCGGG
I5-2	CGGCCCTCGCCTCCCTCA
I5-3	GGGCGTGAGCCCAGACCC
I5-4	TGTCGGCCCAGGACTGCA
I5-5	CCGCCTTCCCCGGAGCTG
I5-6	AGCTCTCCCCAAGGACCT
I5-7	TCCGGCCTGGCTCAGGGC
I5-8	GGGCCCCAGGGTCACCGC
I5-9	GGGCCGGGCCCCAGGGTC
I5-10	CCCGTGGGACACTCTGGG
I5-11	CTCAGAACCAACAGGTGC

Table S2. Lists of primers

Name	Sequence 5'-3'
T7-Fwd	CGACTCACTATAGGGAGACCCA
BgH-Rev	AACAACAGATGGCTGGCAAC
HRAS-Fwd	GAC TCG GAT GAC GTG CCC ATG
Cy5-HRAS-Rev	5'Cy5 - AGG AGG GTT CAG CTT CCG C
GAPDH-Fwd	GGA GCG AGA TCC CTC CAA AAT
GAPDH-Rev	GGC TGT TGT CAT ACT TCT CAT GG
HRAS E4I4 FWD	GCA CGC ACT GTG GAA TCT
HRAS E4I4 REV	GGA GAG CTG CCT CAC CT
HRAS I5 FWD	GGG CAC CTG TTG GTT CT
HRAS I5 REV	CCT CCT GAA CTC CAG GTC T

Table S3. Lists of siRNAs

Name	Sequence 5’-3’	Catalog Number/Reference
siNTC	Silencer Select Negative Control #1	Thermo Fisher 4390843
siHNRNPF	GCGACCGAGAACGACAUUU	(1)
siHNRNPH1	GAAGCAUACUGGUCCAAAU	Thermo Fisher s6728
siHNRNPH/F	GGAAGAAAUUGUUCAGUUC	(21)

Table S4. Lists of antibodies

Name	Manufacturer	Catalog Number
Anti-hnRNP H	Lab made	N/A
Anti-hnRNP F	Lab made	N/A
Anti-hnRNP H/F	Santa Cruz Biotechnology	sc-32310
Anti-His	MBL International	D291-3
Anti-GAPDH	Santa Cruz Biotechnology	sc-47724
AntI-HRAS	Abcam	ab86696
Anti-Flag	Sigma Aldrich	F3165
Mouse IgG isotype control	Invitrogen	14471485
Rabbit IgG isotype control	Thermo Fisher	02-6102
Anti-hnRNP H (IP)	Abcam	ab10374
Anti-hnRNP F (IP)	Sigma Aldrich	04-1462
Anti-MYC	Abcam	ab32072
Anti-cPARP	Cell signaling	5625S
Anti-SRSF5	Sigma Aldrich	HPA043484

Dataset S1 (separate file). Differential SE events after HNRNPH1 KO and HNRNPF KO in HepG2 cells, and in MYC on versus off conditions in the prostate cancer cell model.

SI References

1. M. Nazim, *et al.*, Competitive regulation of alternative splicing and alternative polyadenylation by hnRNP H and CstF64 determines acetylcholinesterase isoforms. *Nucleic Acids Res*, gkw823 (2016).

2. I. Dunham, *et al.*, An integrated encyclopedia of DNA elements in the human genome. *Nature* **489**, 57–74 (2012).

3. A. Dobin, *et al.*, STAR: ultrafast universal RNA-seq aligner. *Bioinformatics* **29**, 15–21 (2013).

4. S. Shen, *et al.*, rMATS: Robust and flexible detection of differential alternative splicing from replicate RNA-Seq data. *Proc. Natl. Acad. Sci. U.S.A.* **111** (2014).

5. L. Demirdjian, *et al.*, Detecting Allele-Specific Alternative Splicing from Population-Scale RNA-Seq Data. *The American Journal of Human Genetics* **107**, 461–472 (2020).

6. P. D. Thomas, *et al.*, PANTHER: Making genome-scale phylogenetics accessible to all. *Protein Science* **31**, 8–22 (2022).

7. J. N. Weinstein, *et al.*, The Cancer Genome Atlas Pan-Cancer analysis project. *Nat Genet* **45**, 1113–1120 (2013).

8. J. Lonsdale, *et al.*, The Genotype-Tissue Expression (GTEx) project. *Nat Genet* **45**, 580–585 (2013).

9. R. L. Grossman, *et al.*, Toward a Shared Vision for Cancer Genomic Data. *New England Journal of Medicine* **375**, 1109–1112 (2016).

10. M. D. Mailman, *et al.*, The NCBI dbGaP database of genotypes and phenotypes. *Nat Genet* **39**, 1181–1186 (2007).

11. J. L. Robinson, A. Feizi, M. Uhlén, J. Nielsen, A Systematic Investigation of the Malignant Functions and Diagnostic Potential of the Cancer Secretome. *Cell Reports* **26**, 2622-2635.e5 (2019).

12. J. Harrow, *et al.*, GENCODE: The reference human genome annotation for The ENCODE Project. *Genome Res.* **22**, 1760–1774 (2012).

13. C. Trapnell, *et al.*, Differential gene and transcript expression analysis of RNA-seq experiments with TopHat and Cufflinks. *Nat Protoc* **7**, 562–578 (2012).

14. J. W. Phillips, *et al.*, Pathway-guided analysis identifies Myc-dependent alternative pre-mRNA splicing in aggressive prostate cancers. *Proc. Natl. Acad. Sci. U.S.A.* **117**, 5269–5279 (2020).

15. A. Liberzon, *et al.*, Molecular signatures database (MSigDB) 3.0. *Bioinformatics* **27**, 1739–1740 (2011).

16. Y. Liao, G. K. Smyth, W. Shi, featureCounts: an efficient general purpose program for assigning sequence reads to genomic features. *Bioinformatics* **30**, 923–930 (2014).

17. M. I. Love, W. Huber, S. Anders, Moderated estimation of fold change and dispersion for RNA-seq data with DESeq2. *Genome Biology* **15**, 550 (2014).

18. H. Han, *et al.*, MBNL proteins repress ES-cell-specific alternative splicing and reprogramming. *Nature* **498**, 241–245 (2013).

19. I. Paz, I. Kosti, M. Ares, M. Cline, Y. Mandel-Gutfreund, RBPmap: a web server for mapping binding sites of RNA-binding proteins. *Nucleic Acids Res* **42**, W361–W367 (2014).

20. F. Piva, M. Giulietti, A. B. Burini, G. Principato, SpliceAid 2: A database of human splicing factors expression data and RNA target motifs. *Human Mutation* **33**, 81–85 (2012).

21. D. Garneau, T. Revil, J.-F. Fisette, B. Chabot, Heterogeneous Nuclear Ribonucleoprotein F/H Proteins Modulate the Alternative Splicing of the Apoptotic Mediator Bcl-x. *Journal of Biological Chemistry* **280**, 22641–22650 (2005).
